# Genome-Wide Identification of Maize Aquaporin and Functional Analysis During Seed Germination and Seedling Establishment

**DOI:** 10.3389/fpls.2022.831916

**Published:** 2022-01-27

**Authors:** Yingchun Su, Zhonghao Liu, Jiahui Sun, Chenglai Wu, Yan Li, Chunqing Zhang, Linmao Zhao

**Affiliations:** State Key Laboratory of Crop Biology, Agronomy College, Shandong Agricultural University, Tai’an, China

**Keywords:** aquaporin, tonoplast intrinsic protein 3, seed germination and seedling emergence, seed vigor, gene family analysis, maize

## Abstract

Water uptake facilitates the initiation of seed germination. It is presumed that aquaporin (AQP)-mediated water inflow contributes to seed germination, but the genetic evidence is still lacking. This study aimed at genome-wide identification of ZmAQPs and further determined the physiological functions. Following a comprehensive search, a total of 41 ZmAQPs were identified according to the latest genome database. Through bioinformatic approaches, the physicochemical characteristics, phylogenetic relationships, and structural features of ZmAQPs were analyzed. The gene expression analysis of 20 high-resolution and multi-tissues samples showed that ZmAQPs had distinct spatiotemporal and tissue-specific expression profiles during seed germination and early seedling development. We then focused on the aquaporin of maize tonoplast intrinsic protein 3 (ZmTIP3), which is specifically expressed in germinating seed. A mutant *zmtip3-1* with disruption of the *ZmTIP3-1* gene showed shorter shoot and root length, and decreased seedling dry weight compared with the control (*W22*). The result revealed that ZmTIP3-1 improved the absolute content of seed protein and promoted storage reserves mobilization, suggesting that ZmTIP3 may be a positive regulator of seed vigor. This work provides valuable clues for understanding the function and possible regulatory mechanism of ZmAQPs in seed germination and seedling growth.

## Introduction

Maize (*Zea mays*) is an important staple food and economic crop worldwide. Seed germination and seedling establishment are crucial initial stages for plant growth and are closely related to the rate and uniformity of field crop establishment, which ultimately determines the yield, especially for maize, in the absence of tillering (Rajjou et al., 2012; Finch-Savage and Bassel, 2016). Germination begins with water uptake by quiescent seed and ends with radicle protrusion, and the subsequent post-germination seedling growth. Seed hydration during germination is displayed in a typical triphasic pattern (Nonogaki et al., 2010; Bewley et al., 2013). Meanwhile, there are substantial physical and metabolic events that occur, including cell expansion, cell division, and reserve mobilization (Nonogaki et al., 2010). However, fundamental research on maize seed germination and early seedling growth is limited.

Water is a prerequisite for germination and is the medium of life activities. Aquaporins (AQPs) are integral membrane proteins that facilitate the movement of water across biological membranes (Chaumont and Tyerman, 2017). Aquaporins exist in membranes with homologous or heterologous tetramer forms (Daniels et al., 1999), and a typical structure of an aquaporin monomer has two conserved NPA (Asn-Pro-Ala) motifs, six membrane-spanning α-helixes (H1-H6), and two short highly hydrophobic α-helixes (HB and HE), with the N- and C-termini exposed to the cytosol (Maurel, 1997; Murata et al., 2000; Chaumont et al., 2001). Based on the subcellular localization and sequence homology, the aquaporin family in higher plants was mainly classified into four subfamilies, including the plasma membrane intrinsic proteins (PIPs), tonoplast intrinsic proteins (TIPs), NOD26-like intrinsic proteins (NIPs), and small basic intrinsic proteins (SIPs) (Chaumont and Tyerman, 2017).

The plant AQP family comprises many members, and their cellular localization diversification and regulatory complexity enables AQPs to function in plant growth and in the response to environmental signals. Therefore, plant AQPs have received increasing attention. With the availability of genomic sequences of plant species, the identification of AQPs provides basic information to better understand the AQPs gene family. Numerous studies have confirmed that plant AQPs can be ubiquitously expressed in all tissues and exhibit clear spatiotemporal-specific patterns at the transcription level (Barrieu et al., 1998; Chaumont et al., 1998; Takahashi et al., 2004; Guo et al., 2006; Vander Willigen et al., 2006; Hunter et al., 2007; Soto et al., 2008; Gattolin et al., 2009; Novikova et al., 2014; Utsugi et al., 2015). Moreover, AQPs are highly regulated by posttranslational modifications, including phosphorylation and heteromerization, that influence the amount and activity of AQPs, trafficking, gating and re-localization, to ultimately control plant water relations (Zelazny et al., 2007; Chaumont and Tyerman, 2014; Maurel et al., 2015; Berny et al., 2016). Few studies have investigated the biological function of AQPs in plant development and growth. The expression of TIP3-1 was detected exclusively in developing seeds and early seed germination and ceased after radicle emergence at the transcriptional level (Johnson et al., 1989; Melroy and Herman, 1991; Vander Willigen et al., 2006; Novikova et al., 2014). In contrast, other AQPs exhibited relatively low expression in dry and germinating seeds. Extensive research has shown that TIP1 and TIP2 substitute for TIP3 after seed germination and retain high expression levels in elongating and dividing tissues (Maurel et al., 1997a; Chaumont et al., 1998; Vander Willigen et al., 2006; Novikova et al., 2014). According to our knowledge, only two previous reports provided genetic evidence that AQPs function in seed germination, that is, *Arabidopsis* AtTIP3 (Footitt et al., 2019) and rice OsPIP1 (Liu et al., 2007) influence seed germination under stress. Water inflow is essential for the initiation of embryonic cell elongation during early germination. Water imbibed by seed maintains cell turgor pressure and causes tissue expansion and subsequent seedling growth; AQPs may be involved in the finely tuned regulation of water transport (Obroucheva et al., 2017). Despite the predicted significance of *TIPs* in seed germination and seedling emergence based on expression analysis, genetic evidence is currently lacking in maize, and the mechanism of the spatial and temporal fine-tuning of *ZmTIPs* remains largely unknown.

Here, we performed a genome-wide characterization of maize aquaporins (ZmAQPs) and a comprehensive analysis of the phylogenetic relationships and physicochemical and structural properties of 41 identified genes. We also investigated the expression profile of AQPs during the seed germination process and then focused on seed-specific ZmTIP3-1. ZmTIP3-1 acts in the regulation of seedling growth and the protein content of the seed. Furthermore, the regulation mechanism of the spatiotemporal-specific pattern of ZmTIPs during seed germination is discussed in depth.

## Materials and Methods

### Materials

The maize inbred lines B73 and Z58 (Zheng58), with different genetic backgrounds, were used to investigate the expression patterns of ZmAQPs during seed germination and seedling emergence. For the physiological function analysis, the UniformMu insertion mutant *zmtip3-1* (UFMu-13193) was obtained from the Maize Genetics Cooperation Stock Center. The *zmtip3-1* mutant was backcrossed five generations with *W22* and then selfed to generate BC5F2, from which homozygous *zmtip3-1* was identified for the phenotype analysis of seed germination and seedling growth. All the maize materials were grown at the Experimental Station of Shandong Agricultural University.

### Identification of Maize Aquaporins and Bioinformatic Analysis

Two methods were used to identify the members of the ZmAQP gene family. The Hidden Markov Model (HMM) file corresponding to the MIP domain (PF00230) was downloaded from the PFAM database.^1^ HMMER (3.3.1) was used to search for AQP proteins from the maize genome database. The AQP protein sequences of *Arabidopsis* and rice reported in previous research (Quigley et al., 2001; Sakurai et al., 2005) were downloaded from the UniProt database.^2^ These protein sequences were used as queries in BLASTP searches with BLASTP suite (blast 2.5.0). The latest maize genome information, Zm-B73-REFERENCE-NAM-5.0 (GCF_902167145.1), was retrieved from NCBI.^3^ Multiple sequence alignment and phylogenetic trees were analyzed by MEGA-X (Sudhir et al., 2018), and the trees were visualized by FigTree (v1.4.4). The specific chromosomal positions and collinearity of AQPs were processed with MapInspect software and TBtools (MCScanX) (Chen et al., 2020). Basic physical and chemical characteristics, the subcellular localization, transmembrane domains, gene structures, motifs, *cis*-acting regulatory elements and the phosphorylation site of AQPs were determined by online websites, as follows: ProtParam tool,^4^ WoLF PSORT^5^ and TMHMM Server v.2.0,^6^ Gene Structure Display Server (GSDS 2.0) (Hu B. et al., 2015), MEME web server (Bailey et al., 2009), plantCARE database (Lescot et al., 2002) and NetPhos 3.1,^7^ respectively. The 3D structure was predicted by the phyre2.0 website^8^ and visualized by VMD software (Kelley et al., 2015). The interaction network for ZmAQPs was searched from the string site^9^ and displayed through CytoScope software (Shannon et al., 2003).

### Subcellular Localization Assays

The full-length cDNAs of ZmAQPs were subcloned into the pSAT6-EYFP_N1 vector to create the ZmAQP-YFP constructs. The protoplast isolation and transient expression were conducted as described previously (Tu et al., 2020). Mesophyll protoplasts were isolated using the 7-d-old seedling leaves under dark condition. The ZmAQP-YFP fusion plasmid was transfected into protoplasts using the PEG-calcium solution (0.4 g/mL PEG 4000, 0.8 M mannitol, 0.1 M CaCl_2_). After being washed and resuspendeding with W5 solution (154 mM NaCl, 125 mM CaCl_2_, 5 mM KCl, 2 mM MES), mesophyll protoplasts were incubated under dark for 12–18 h. The YFP fluorescence was examined and imaged with a confocal microscope.

### Seed Germination Test and Phytohormone Treatment

The standard germination test was performed as described previously (Zhao et al., 2020). The trials were conducted in sand with three 50-seed replicates for each material. Radicle emergence characteristic was recorded every 4 h after imbibition. Radicle emergence is defined as radicle > 2 mm long. The seedlings were allowed to grow for 7 days and the number of germinated seeds was counted at 24 h-intervals. Germination index (GI) and seed vigor index were calculated, GI = Σ (Gt/Dt); VI = GI × W, where Gt is the number of germinated seeds on day, t and Dt is time in days, W is the dry weight per seedling (g). For recording dry weight, all normal seedlings were dried at 105°C for 30 min and then transferred to 80°C and dried until constant weight.

The samples used for gene expression profiles analysis were obtained from the process of germination and post-germination, including the imbibed seeds, root and shoot at different stages, and 4 days seedlings (mesocotyl, plumule, and coleoptile included). Different zones of primary maize root were collected according to previous studies (Kozlova et al., 2012; Yu et al., 2016). To determine the effects of phytohormones on ZmAQPs, we set three groups of treatments: dry seeds were imbibed in the solutions of 10 μM ABA (Solarbio, A8060) and 10 μM GA (Solarbio, G8040); 10 μM IAA (Aladdin, I101074), and 10 μM ethephon (Solarbio, E8020) were spared on the germinated seeds (just after radicle emergence) and seedlings (just growing out of the soil), respectively. Water treatment was used as a control. All the samples for analysis were collected 3 h after treatment.

### Gene Expression Analysis

Total RNA was extracted using the TIANGEN RNA Extraction Kit (DP445) according to the manufacture’s protocol. The first-strand cDNA was synthesized using the PrimeScript™ RT reagent Kit (Takara, RR047A). RT-qPCR analysis was performed using the TB Green^®^ Premix Ex Taq™ (Takara, RR420A) on QuantStudio™ 12K Flex Software (Thermo Fisher Scientific). Each sample was quantified at least in triplicate and normalized using *ACTIN 1* as a reference gene. The primer sequences of *ACTIN 1* used were described previously (Zhang Z. et al., 2015). The gene-specific primer pairs for RT-qPCR are listed in Supplementary Table 5.

### Western Blot

Protein expression levels of *zmtip3-1* mutant and *W22* were determined by western blotting. Seeds imbibed for 0, 12, and 24 h of both *zmtip3-1* and *W22* were harvested for analysis. Total proteins were extracted in an extraction buffer (50-mM Tris-HCl at pH 7.5, 150-mM NaCl, 1 mM EDTA, 10 mM NAF, 25 mM β-glycerophosphate, 2 mM sodium orthovanadate, 10% triglyceride, 0.1% tween-20, 0.6% SDS, 1 mM DTT, and 1 mM PMSF) containing protease inhibitor cocktail (Roche). The proteins were detected by SDS-PAGE gel with an anti-TIP3 antibody, which was produced using a synthetic peptide specific for maize aquaporin TIP3 (Abmart). Anti-Actin antibody (CW0096, Abmart) was used as a control.

### Statistical Analysis

The data were analyzed by Student’s *t*-test using SPSS 19.0 software (SPSS, Inc., Chicago, United States).

## Results

### Genome-Wide Identification of Aquaporins in Maize

A comparison of HMMER and BLASTP searches of the *Z. mays* genome identified 43 putative ZmAQP sequence items. Based on the predicted conserved domains and annotation of the sequences, duplicate sequences were discarded. We finally obtained 41 complete and unique genes encoding ZmAQPs. Despite 41 ZmAQPs being identified in the work of Bari et al. (2018), there were errors, duplicate genes, and confusing nomenclature. For example, the ZmTIP4;1 and ZmTIP4;1a were in the same gene ID (Zm00001d037779) by searching the maize genome B73 RefGen_v4. Besides, ZmTIP4; 1a was named as ZmTIP4-1 by Chaumont et al. (2001). Here, *ZmAQP* genes were named with reference to homologous genes of Arabidopsis and rice, and of which 30 genes were annotated by Chaumont et al. (2001). The ZmAQP names, gene ID, protein length, molecular weight (MW), and theoretical isoelectric point (PI) are shown in Table 1. Compared with the work of Chaumont et al. (2001), two genes, *zmpip1-4* and *zmpip1-6*, were removed, while eleven novel identified genes were added (Table 1). Initially, Chaumont et al. (2001) noted that *zmpip1-3* and *zmpip1-4* present in the B73 inbred line encoded the same protein with different nucleotide sequence. We confirmed that the *zmpip1-3* and *zmpip1-4* had the same accession (NP_001352636) and the same gene ID (Zm00001eb186900) in Zm-B73-REFERENCE-NAM-5.0 genome database. For *zmpip1-6*, was not in the Zm-B73-REFERENCE-NAM-5.0 but was in B73 RefGen_v4 with the gene ID Zm00001d020383. Multiple protein sequence alignments of the 41 identified ZmAQPs are presented in Supplementary Figure 1. All of the ZmAQPs contained two conserved NPA motifs and some highly conserved domains or sites (Supplementary Table 1). The NPA motifs were well conserved, by contrast, ZmNIP3-1, ZmNIP4-1, ZmSIP1s, and ZmSIP-1 encoded motifs with a variable third residue in which A was replaced by either I, L, T, or V (Supplementary Table 1).

**TABLE 1:** Aquaporin genes in the *Z. mays* genome.

Gene name	NCBI accession	Gene ID^a^	AA count^b^	MW (KDa)	PI	Subcellular localization^c^
						
ZmPIP1-1	NP_001105466	Zm00001eb074210	288	30.7	9	Plas
ZmPIP1-2	NP_001104934	Zm00001eb249940	289	30.8	9	Plas
ZmPIP1-3	NP_001352636	Zm00001eb186900	292	31.0	8.83	Plas
ZmPIP1-5	NP_001105131	Zm00001e023558	288	30.7	8.3	Plas
ZmPIP2-1	NP_001105024	Zm00001eb306380	290	30.2	7.69	Plas
ZmPIP2-2	NP_001105638	Zm00001eb096680	292	30.3	8.29	Plas
ZmPIP2-3	NP_001105025	Zm00001eb185300	289	30.4	6.95	Plas
ZmPIP2-4	NP_001105026	Zm00001eb247760	288	30.3	6.5	Plas
ZmPIP2-5	NP_001105616	Zm00001eb077130	285	29.8	7.7	Plas
ZmPIP2-6	NP_001105027	Zm00001eb306400	288	30.2	8.38	Plas vacu
ZmPIP2-7^n^	XP_020397030	Zm00001eb331830	288	30.3	8.82	Plas vacu
ZmPIP2-8^n^	NP_001310831	Zm00001eb223910	283	30.1	9.19	Plas
ZmPIP2-9^n^	XP_008670063	Zm00001eb096620	286	29.8	8.38	Plas vacu
ZmTIP1-1	NP_001104896	Zm00001eb003730	250	25.8	6.02	Plas vacu
ZmTIP1-2	NP_001105029	Zm00001eb362170	254	25.4	5.87	Plas vacu
ZmTIP2-1	NP_001105030	Zm00001eb186570	249	24.9	5.3	Plas vacu
ZmTIP2-2	NP_001105031	Zm00001e015330	250	25.0	5.59	Plas vacu
ZmTIP2-3	NP_001358543	Zm00001eb429750	248	25.1	6.16	Vacu plas
ZmTIP2-4^n^	XP_008669054	Zm00001eb074660	248	25.0	5.87	Plas vacu
ZmTIP3-1	NP_001105032	Zm00001eb221090	262	27.2	8.12	plas
ZmTIP3-2	NP_001105045	Zm00001eb044880	266	27.4	8.13	Plas
ZmTIP3-3^n^	NP_001146930	Zm00001eb404170	267	27.3	9.79	Plas
ZmTIP3-4^n^	XP_008669132	Zm00001eb076690	265	27.5	9.05	Plas vacu
ZmTIP4-1	NP_001105033	Zm00001eb284350	255	26.5	6.7	Vacu plas
ZmTIP4-2	NP_001105034	Zm00001e027152	311	32.3	7.9	Plas vacu
ZmTIP4-3	NP_001334625	Zm00001eb123230	249	25.2	6.42	Plas vacu
ZmTIP4-4	NP_001105641	Zm00001eb119200	252	25.3	6.42	Vacu plas
ZmTIP5-1	NP_001105036	Zm00001eb429760	260	26.5	7.74	Plas
ZmNIP1-1	NP_001105721	Zm00001eb239170	282	29.6	8.58	Plas
ZmNIP1-3^n^	NP_001151947	Zm00001eb283960	284	29.4	7.67	Plas vacu
ZmNIP1-4^n^	XP_008660914	Zm00001eb384790	284	30.1	6.07	Plas vacu
ZmNIP2-1	NP_001105637	Zm00001eb254540	295	31.8	6.79	Plas
ZmNIP2-2	NP_001105020	Zm00001eb279660	294	31.4	7.71	Plas cyto_plas
ZmNIP2-3	NP_001105517	Zm00001eb372380	301	31.7	7.04	Plas ER
ZmNIP2-4^n^	NP_001131324	Zm00001eb194530	303	32.3	6.39	Plas ER
ZmNIP3-1	NP_001105021	Zm00001eb043650	302	31.2	9.22	Plas vacu
ZmNIP3-2^n^	XP_008664035	Zm00001eb416440	274	27.7	6.97	Vacu
ZmNIP4-1^n^	NP_001354974	Zm00001eb126240	299	31.1	8.2	Plas
ZmSIP1-1	NP_001105514	Zm00001eb167790	245	25.6	8.43	Vacu
ZmSIP1-2	NP_001105028	Zm00001eb349840	243	25.7	9.35	Vacu plas
ZmSIP2-1	NP_001105640	Zm00001eb015230	249	26.8	9.86	Plas

*Aquaporin genes in the Z. mays genome. Gene-related information, physicochemical properties (amino acids count, molecular weight, pI, chromosomal location) and the predicted subcellular localization of the identified ZmAQPs are presented. The annotation of some items is listed at the bottom.*

*^a^Gene IDs are based on Zm-B73-REFERENCE-NAM-5.0.*

*^b^Number of amino acids (AA).*

*^c^Predicted subcellular localization of ZmAQPs by the WOLF PSORT program. plas, plasma membrane; vacu, tonoplast; cyto, cytosol; ER, endoplasmic reticulum.*

*^n^Newly identified ZmAQPs.*

Protein domain prediction analysis demonstrated that all of the sequences contained putative transmembrane α-helices but showed differences in the number of transmembrane domains. Most AQP proteins have a typical structure with six membrane-spanning domains, and five ZmAQPs contain five or seven (Supplementary Table 1). As a highly reliable means, the 3D structure prediction was further conducted for the prediction of the tertiary structure of the five ZmAQPs maps. 3D structural modeling revealed that each aquaporin monomer was formed by six long helices (namely TM1-TM6, with two on the surface and four at the bottom of the picture) and two re-entrant short helices (HB and HE, facing the readers) (Supplementary Figure 2). Hence, we considered that all 41 ZmAQPs had six transmembrane domains.

**FIGURE 1:**
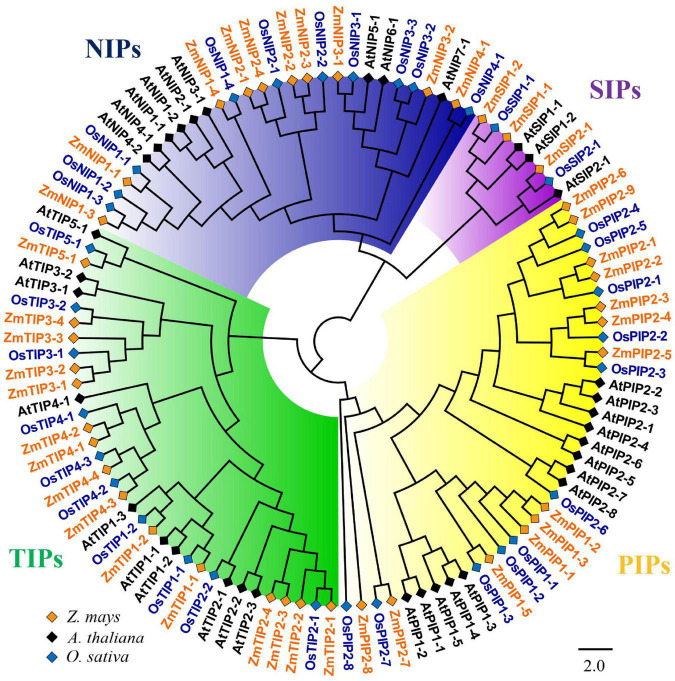
Phylogenetic analysis of 109 aquaporin proteins from *Z. mays* (Zm, indicated in orange), *A. thaliana* (At, indicated in black), and *O. sativa* (Os, indicated in blue). The ZmAQPs clustered into four different subfamilies (PIPs, TIPs, NIPs, and SIPs), with the corresponding OsAQP and AtAQP subfamilies. Each AQP subfamily is highlighted with a specific background color. PIPs, TIPs, NIPs, and SIPs are indicated in yellow, green, blue and purple, respectively. Multi-species protein sequence alignment was performed using Clustal W software. The phylogenetic tree was constructed using MEGA X based on the NJ method and further improved using the FigTree (v1.4.4) software.

To characterize the phylogenetic relationships and to predicate the putative function of the ZmAQPs, a phylogenetic tree of 109 AQP sequences was constructed among *Arabidopsis*, rice, and maize. Phylogenetic analysis showed that ZmAQPs clustered into four different subfamilies (PIPs, TIPs, NIPs, and SIPs), the same as OsAQPs and AtAQPs (Figure 1). Among the 41 identified ZmAQPs proteins, 13 belong to PIPs, 15 to TIPs, 10 to NIPs, and 3 to SIPs. The phylogenetic analysis and subcellular localization of ZmAQPs contribute to further understanding or predicting their functional properties. According to the predicted subcellular localization, ZmAQP subfamilies exhibited distinct subcellular localizations and certain preferences. The PIPs are predominately localized to the plasma membrane, while most of the TIPs members (TIP1s, TIP2s, and TIP4s) are located at the plasma membrane and vacuolar membrane (tonoplast) (Table 1). Dual localization of plant AQPs was found in previous studies, including ZmPIP1-2 and AtTIP3s (Chaumont et al., 2000; Gattolin et al., 2011), suggesting versatile roles of AQPs. ZmTIP3-1 and TIP3-2 were predicted to localize to the plasma membrane (Table 1). In accordance with the prediction analysis, subcellular localization of ZmTIP3s and some other AQPs was mainly on the plasma membrane (Supplementary Figure 3). All observations showed that the complexity of AQP subcellular localization, which is compatible with the AQP functional diversity.

### Structural Characterization and Evolutionary Analysis of the Maize Aquaporin Gene Family

**FIGURE 2:**
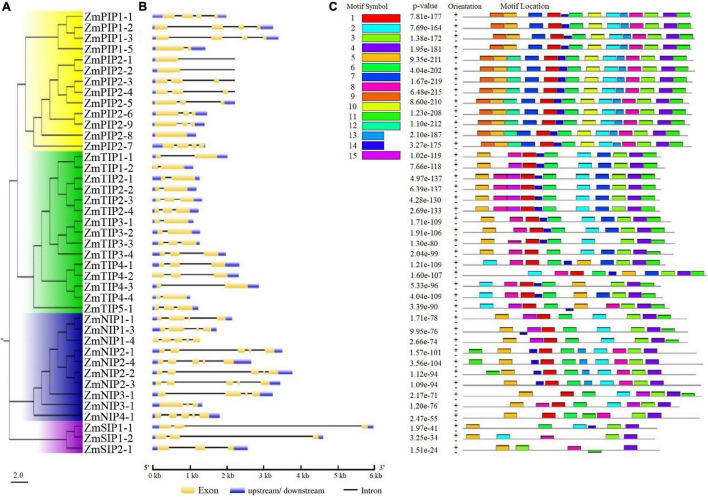
Phylogenetic relationships, gene structure and motif compositions of ZmAQPs. **(A)** The neighbor-joining evolutionary tree of 41 ZmAQP protein sequences constructed using MEGA X software. The details of four subfamilies are shown in different background colors. **(B)** Exon-intron gene structure of ZmAQP genes. Exons (CDS) and introns are represented by yellow boxes and black lines, respectively. Blue boxes indicate the upstream or downstream regions of genes. Gene length is shown in kb on the x-axis. **(C)** Distribution of conserved motifs in ZmAQPs. The conserved motifs were identified using the MEME online program. Different motifs, numbers 1–15, are highlighted with different colors. The sequence information for each motif is provided in Supplementary Table 2.

The exon-intron organizations of all identified *ZmAQP* genes were examined. The gene structure analysis showed that the exon-intron counts and distribution in the *ZmAQP* genes, varied from one to five exons per gene (Figure 2B and Supplementary Table 1). The exon-intron distribution and length were closely coincident with the alignment clusters of *ZmAQP* genes (Figure 2). The variation in the *ZmAQP* gene structure was also observed in the four subfamily members. Overall, the gene structure was diverse in the ZmAQP gene family but was relatively conserved in each subfamily. The differences in gene structure might confer functional variations. We further identified 15 conserved motifs (designated as motif 1 to motif 15, Supplementary Table 2) in the ZmAQP family to gain more insight into the ZmAQP diversity. As shown in Figure 2C, ZmAQP members within the same subfamily were usually found to share a similar motif composition. The differences among ZmAQP subfamilies were reflected in the motif number and their distribution (Figure 2C). The PIPs possess the most motifs and the motif arrangements are regular with specific ordering and similar positions. In summary, the similar gene structure and highly conserved protein motifs within the same subfamily were in agreement with the phylogenetic analysis, indicating the reliability of the above analysis. As shown in Supplementary Figure 4, the identified 41 *ZmAQP* genes were unevenly distributed among 10 chromosomes according to the number of genes on each chromosome. The distribution of *ZmAQP* genes in the maize genome could be affected by some genetic events such as segmental duplication (Lynch and Conery, 2000). The result indicated that 11 segmental duplication events within 15 *ZmAQP* genes were identified. Furthermore, the duplication events between genes usually occurred on chr2, chr4, and chr5 (Supplementary Figure 4). The tandem duplication events were also attributed to gene family evolution. Duplications of individual genes or chromosomal segments may provide an idea for research directions regarding new gene functions and expression patterns.

### Analysis of the Spatial-Temporal Expression Patterns of Maize Aquaporins

To elucidate the spatial-temporal pattern of the ZmAQPs, we reanalyzed the publicly available RNA-seq data of 79 samples in different developmental stages, including roots, internodes, leaves, young stems, reproductive organs, and seeds (Stelpflug et al., 2015). As shown in Supplementary Table 3, most of the *PIPs* had high expression in all the samples examined, indicating a constitutive expression profile during plant development. Notably, *PIPs* showed relatively higher expression levels in roots, with relatively low expression levels in the other tissues especially the developing seeds. Indeed, it is not difficult to understand the results, as root is the primary organ for water uptake and the general role of aquaporins is to regulate transmembrane water transport (Peret et al., 2012; Chaumont and Tyerman, 2017; Ding et al., 2020).

Meanwhile, *ZmTIP1-2* showed higher expression in the root and anthers, *ZmTIP2s* mainly in the roots, and *ZmTIP4s* in the leaves. The result also proved that *ZmTIP3s* are seed-specific and are exclusively expressed in developing seed and early germination stages. Overall, the RNA-seq analysis suggests that the expression profile of *ZmAQPs* varies with tissues and developmental stages.

**FIGURE 3:**
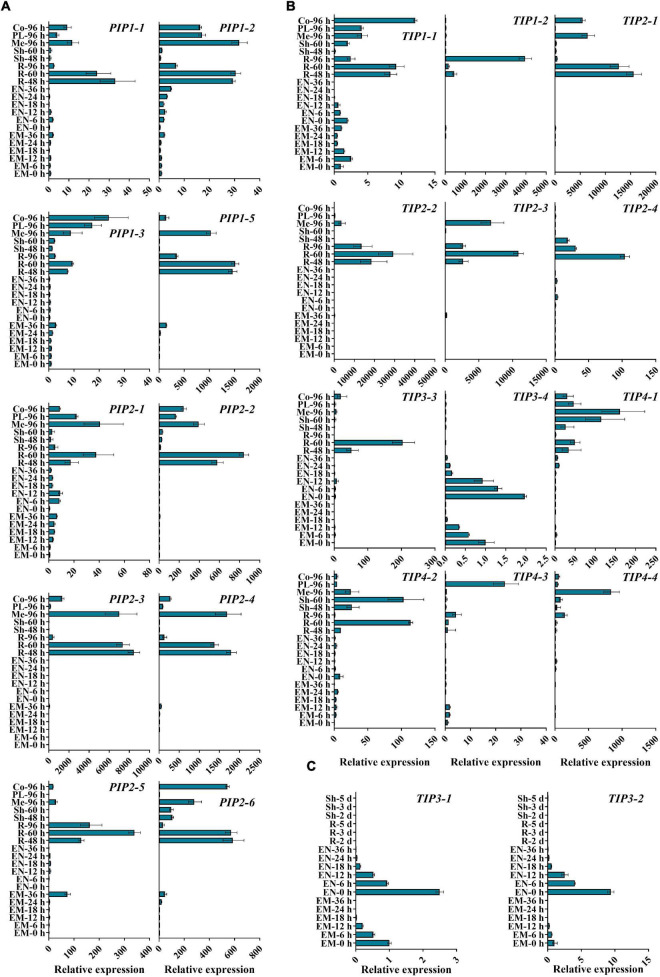
qRT-PCR analysis of the expression levels of *ZmAOPs* during early seed germination. The relative transcript levels of *ZmPIPs* **(A)** and Zm*TIPs* **(B,C)** in B73 during the seed germination process. Twenty samples from seeds and seedlings at different times after seed imbibition were used in the assay. *ACTIN 1* was used as an internal control. Data are means ± *SD* (*n* = 3). Three independent experiments produced similar results. EM, embryo; EN, endosperm; R, root; Sh, shoot; Me, mesocotyl; PL, plumule; Co, coleoptile.

**FIGURE 4:**
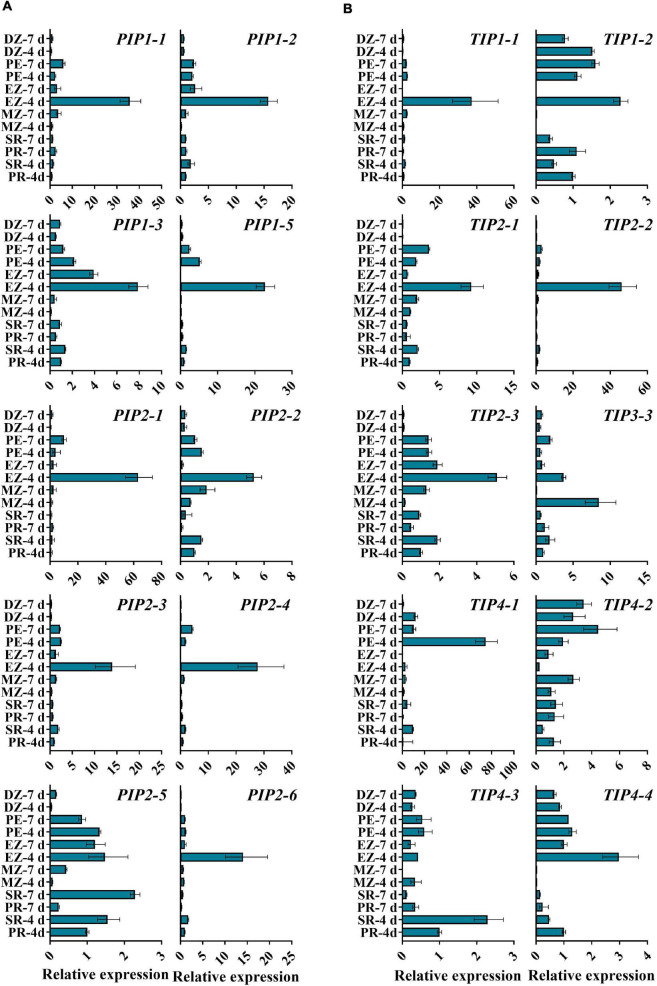
qRT-PCR analysis of the expression levels of *ZmAOPs* in different zones of the seedling root. The relative transcript levels of *ZmPIPs* **(A)** and Zm*TIPs* **(B)** in B73. Samples from roots at 4 or 7 days after seed imbibition were used in the assay. *ACTIN 1* was used as an internal control. Data are means ± *SD* (*n* = 3). PR, primary root; SR, secondary root; MZ-root cap and meristem zone of the primary root; EZ-elongation zone of the primary root; PZ-post elongation zones of the primary root; DZ-differentiation zone of the primary root.

Despite the vital roles in plant development and stress responses, the function of AQPs in the process of seed germination and seedling morphogenesis remains largely unclear. Next, we explored the expression patterns of *ZmPIPs* and *ZmTIPs* during seed germination and early seedling growth using qRT-PCR. Here, we applied a high temporal/spatial-resolution analysis using 20 samples collected within the first 96 h after B73 seed imbibition, including the embryo and endosperm at different times after seed imbibition and the root and shoot (mesocotyl, plumule, and coleoptile) at different stages of seedling growth. In the embryo of imbibed seeds, there was a clear trend toward increasing *PIP* transcript accumulation, peaking in the seeds imbibed for 36 h (EM-36 h, radicle protrusion) (Figure 3A). In post-germination seedlings, all the *PIPs* genes were ubiquitously expressed in the root, mesocotyl, or coleoptile, with significantly higher expression levels in seedlings than in imbibed seeds (Figure 3A). Because of the function of roots in water uptake and because most ZmAQPs showed the highest transcript abundances in the root (Figure 3), the transcriptional levels of some selected *PIP* genes in different zones of the root were also investigated. Almost all of the *PIPs* examined showed the highest expression in the rapid elongation zones of roots at 4-d-old seedlings (EZ-4 d), as opposed to 7-d-old seedlings (Figure 4A).

The transcripts of *TIP* genes exhibited tissue- and stage-specific profiles of accumulation (Figures 3B,C). High expression levels of *TIP3s* (*TIP3-1*, *TIP3-2*, and *TIP3-4*) were observed in dry and imbibed seeds, but the transcripts of *TIP3s* disappeared upon completion of germination (Figure 3C). In contrast, prevalent expression of *TIP1*, *TIP2*, and *TIP4* subgroup members was induced hundreds and thousands of times after radicle emergence (Figure 3B). The expression patterns of *TIPs* in different root zones are in general agreement with those of *PIPs* (Figure 4). Several genes displayed different expression patterns. *PIP2-5* and *TIP1-2* were detected in several zones of roots at different stages, and there were few significant overall differences among the samples (Figure 4). Similar to *TIP4-1*, the expression levels were higher in the PE (post-elongation zones) compared with the other root zones (Figure 4). The results of the ZmAQP expression profiles remained largely similar to those observed in the Z58 line (Supplementary Figures 5, 6). Our qRT-PCR analysis indicated that *TIP1*/*TIP2* progressively substituted for *TIP3* after radicle protrusion and remained at relatively high expressed levels during early seedling development, consistent with previous research (Maurel et al., 1997a; Novikova et al., 2014). Together, in the maize seed germination and post-germination processes, our results revealed a strong link between *ZmAQP* expression and tissue expansion (Figures 3, 4).

The elongation of the radicle and mesocotyl is the key event for maize seed germination and seedlings growing out of the soil (Kutschera and Wang, 2016). These processes are closely related to AQP-mediated water uptake and transport, which maintain cell turgor and cause cell elongation (Maurel et al., 1997a; Obroucheva et al., 2017; Wang et al., 2020). Considering the seed-specific TIP3s during early germination, we first validated the biological function of TIP3s in subsequent experiments.

### Maize Tonoplast Intrinsic Protein 3 Enhances Maize Seed Vigor and Improves Seedling Growth

**FIGURE 5:**
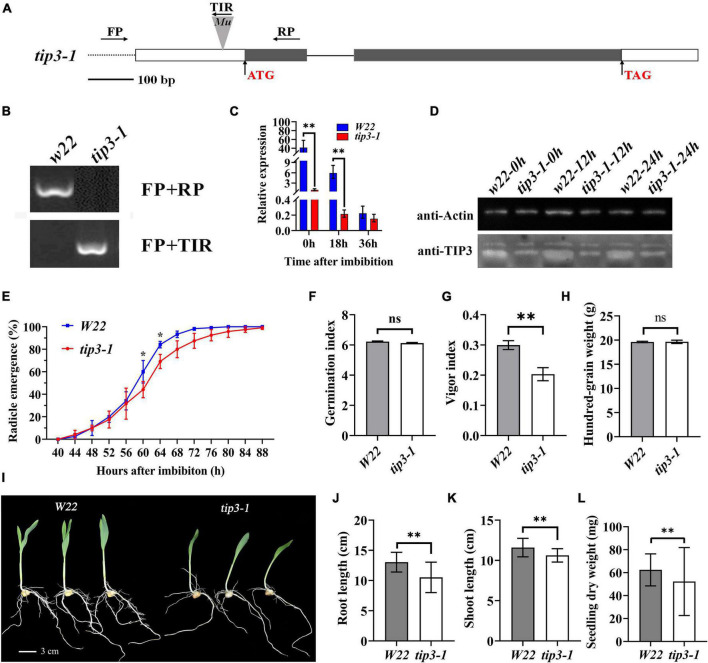
Phenotypic analysis of the *zmtip3-1* mutant. **(A)** Gene structure of the ZmTIP3-1 and Mu insertion sites. Exons are shown as black boxes and introns as lines. The 5’ UTR and 3’ UTR are shown as white boxes. The Mu insertion site and primer sites are indicated. **(B)** Identification of the mutant by PCR at the genome level. **(C)** qRT–PCR analysis of *ZmTIP3-1* transcript abundance in *W22* and *zmtip3-1* seeds. The samples were collected at 0, 18, and 36 h after imbibition. *ACTIN 1* was used as an internal control. Data are means ± *SD*. **(D)** Immunoblot analysis of anti-TIP3 in *zmtip3-1* and *W22*. Anti-actin antibody was used as the internal control. Total proteins were extracted from whole seeds at 0, 12, and 24 h after imbibition. **(E)** Comparison of the germination rate between *zmtip3-1* and *W22*. Radicle emergence characteristics were recorded every 4 h after imbibition. Radicle emergence is defined as radicle length > 2 mm. Data are means ± *SD* (*n* = 3). There were three biological replicates and each included 100 grains. Comparison of the germination index (GI, **F**), vigor index (VI, **G**), and 100-grain weight **(H)** between *zmtip3-1* and *W22*. Seedling growth **(I)**, root length **(J)**, shoot length **(K)** and seedling dry weight **(L)** in *zmtip3-1* and *W22* at 7 days after imbibition. * and ^**^ indicate the significant differences at 5 and 1% levels, respectively, according to Student’s *t*-test, ns indicates non-significant.

To determine the role of ZmTIP3-1 in seed germination, a *Mu-*inserted *zmtip3-1* mutant line was obtained from the Maize Genetics Stock Center. The uniform Mu was inserted into the 5′UTR (43 bp upstream from ATG) of the *ZmTIP3-1* gene (Figure 5A). We first crossed the *zmtip3-1* mutant into the *W22* background and backcrossed 5 generations with *W22* to generate BC5F1, followed by selfing, from which a homozygous *zmtip3-1* line with the *Mu* locus was identified by PCR analysis for subsequent studies (Figure 5B). Furthermore, the qRT-PCR analysis demonstrated that *Mu* insertion resulted in complete inhibition of ZmTIP3-1 expression in the *zmtip3-1* mutant at different times after imbibition (Figure 5C). With the western blot analysis, the protein accumulation level of ZmTIP3-1 was significantly lower in the *zmtip3-1* mutant compared with *W22* before radicle protrusion (Figure 5D). A standard seed germination test disclosed that relative to the control, mutation of ZmTIP3-1 was essentially unchanged in terms of radicle emergence or germination index (GI, which reflects germination speed and uniformity) (Figures 5E,F). There were no differences in the 100-grain weight between the mutant and *W22* (Figure 5H). Based on these results, ZmTIP3-1 minimally influences the seed germination rate and does not regulate seed size and weight. However, as shown in Figure 5G, *zmtip3-1* showed a significant decrease in the vigor index (VI), which is an important index used to evaluate seed quality; VI determines the rate and uniformity of seed germination, and seedling growth (ISTA, 2014). Phenotypic analysis indicated that disruption of ZmTIP3-1 resulted in weaker seedlings (Figure 5I). The growth of 7-d-old mutant seedlings was significantly reduced compared with that of *W22* according to the length and dry weight of the seedlings (Figures 5J–L). Accordingly, our results demonstrated that disruption of ZmTIP3-1 significantly lowered the vigor index mainly by reducing the seedling growth.

Based on the temporal and spatial expression patterns of *TIP3-1*, there was an observation argues this it could be related to preparation for germination (Obroucheva and Sin’kevich, 2010). Our results corroborated this idea. The study further attempted to investigate the underlying mechanisms.

### Maize Tonoplast Intrinsic Protein 3-1 Influences the Absolute Content of Protein in Maize Seeds and Storage Reserves Mobilization

**FIGURE 6:**
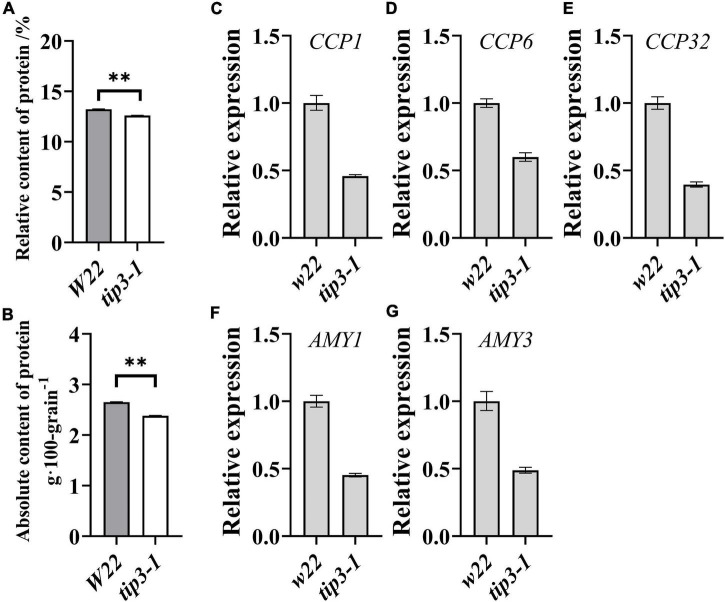
Protein content and mobilization-related genes expression in seeds. Determination of the relative protein content **(A)** and the absolute protein content per 100 grains **(B)** in *tip3-1* mutants and *W22*. All the seeds were harvested at maturity. We multiplied the relative content of the components by the 100-grain weight to calculate the absolute content of the components (g⋅100 grain^–1^). Data are means ± SD (*n* = 3). qRT-PCR analysis of the expression levels of mobilization-related genes of *cysteine protease1 (CCP1*, **C***), cysteine protease 6 (CCP6*, **D***), cysteine protease 32 (CCP32*, **E***), alpha amylase 1 (AMY1*, **F***), alpha amylase 3 (AMY3*, **G**). The samples were collected at 36 h after imbibition. *ACTIN 1* was used as an internal control. Data are means ± *SD*. ** indicates the significant differences at 1% level, according to Student’s *t*-test, ns indicates non-significant.

In maize, the accumulation of *ZmTIP3-1* transcripts increased with maize kernel development and was highest at the mature stage (Supplementary Figure 7). Notably, the expression of *ZmTIP3-1* in the dry seed was higher than that of other AQPs (Supplementary Figure 8). TIP3-1, as a representative aquaporin in PSV, is highly conserved in higher plants (Johnson et al., 1989; Chaumont and Tyerman, 2017). Previous research determined that the abundance of TIP3-1 was not correlated with the content of storage protein in the cotyledon of protein-rich soybean seed (Johnson et al., 1989; Melroy and Herman, 1991). Our previous study verified that the absolute content of protein was significantly correlated with the seedling dry weight of maize and wheat seeds (Wen et al., 2018). However, the correlation between TIP3 accumulation and the protein content in starch-rich cereal seeds remains unclear. To this end, the impact of *ZmTIP3-1* on the grain protein content needs to be evaluated. In the present study, we detected a significant decrease in the absolute content of protein in *zmtip3-1* mutant seeds compared with the W22 (Figures 6A,B). Collectively, we considered that ZmTIP3-1 has a desirable effect on the absolute content of protein of maize seed, which might result in reduced seedling growth of the *zmtip3-1* mutant.

The mobilization of seed storage reserves is one of the major events in germination and seedling growth. We determined the expression levels of genes related to storage reserves mobilization in the *zmtip3-1* mutant. Compared with the *W22*, the expression levels of starch metabolism-related genes, alpha amylase 1 (AMY1) and alpha amylase 3 (AMY3), and protein degradation related genes, cysteine protease1 (CCP1), cysteine protease 6 (CCP6), cysteine protease 32 (CCP32), are significantly decreased in the *zmtip3-1* mutant (Figures 6C–G), suggesting that ZmTIP3-1 enhances seedling growth may be by promoting storage reserves mobilization.

## Discussion

### Spatiotemporal Expression of Maize Aquaporins During Seed Germination and Seedling Emergence

Water uptake mediates the initiation of germination and sequential metabolic activation (Nonogaki et al., 2010), in which AQPs accumulate and are actively involved (Obroucheva et al., 2017; Hoai et al., 2020). For maize, immediately after radicle emergence, the expressions of large members of ZmAQPs are activated in rapid expansion tissues, such as the root elongation zone and elongated mesocotyl, indicating the roles of AQPs in seedling emergence (Figures 3, 4 and Supplementary Figures 5, 6). The growing tissues require a constant water supply for ensuring the increased turgor pressure upon cell enlargement (Obroucheva and Sin’kevich, 2010). Based on the functions of AQPs, PIPs probably mainly act on water inflow and cell-to-cell water transport, whereas TIPs function in maintaining turgor pressure (Trofimova et al., 2001; Javot and Maurel, 2002). Consistent with previous findings of dual localization of AtTIP3s (Gattolin et al., 2011), our results suggest that ZmTIPs can localize in the plasma membrane (Table 1 and Supplementary Figure 3). Despite the significantly higher expression level of ZmTIP3s compared with other ZmAQPs in dry and imbibed seed (Figure 3 and Supplementary Figure 8), plasma membrane localization of ZmTIP3s may ensure effective water absorption during early germination. Compared with the dry seed, most of ZmTIP1s and TIP2s had up to 10,000-fold greater expression post-germination, which was significantly higher than the magnitude of the increase in ZmPIPs (Figures 3A,B and Supplementary Figures 5A,B). In addition, *in vitro* experiments showed water permeability and aquaporin activity of the tonoplast were higher than those of the plasma membrane (Maurel et al., 1997b; Niemietz and Tyerman, 1997; Trofimova et al., 2001). This would help to explain the dramatic changes in TIP expression after germination. *Arabidopsis* AtTIP1 and AtTIP2 have been confirmed to promote cell elongation (Reinhardt et al., 2016). Given the roles of the vacuole in plant cells and dual localization, ZmTIPs could participate in water inflow into the cells and water exchange between the vacuole and the cytoplasm for maintaining turgor to meet early seedling growth.

In contrast to full-scale activation of other ZmTIPs post-germination, ZmTIP3s (including TIP3-1, TIP3-2, and newly characterized TIP3-4) were distinctive (Figure 3C and Supplementary Figure 5C). Since the first identification of α-TIP (TIP3) in plants, the expression profile had been unequivocally determined, to be seed-specific with high expression in maturing and germinating seed, and almost completely abolished after germination (Johnson et al., 1989). Our results (Figure 3C and Supplementary Figure 9) aligned with previous experiments and confirmed this finding. Although it was presumed that TIP3 contributed to seed germination, this hypothesis was not validated by functional analysis until 2019, i.e., that AtTIP3 influences seed germination under water stress (Footitt et al., 2019). We established that ZmTIP3-1 had no desirable effects on germination rate but did affect maize seedling growth (Figure 5). Additionally, the accumulation of TIP3 was opposed to that of TIP1s (TIP2s) at the transcript level, which raised great concern (Maurel et al., 2008; Chaumont and Tyerman, 2017). There was a direct correlation between the disappearance of TIP3 and the coalescence of protein storage vacuoles (PSVs) (Lee et al., 2015, 2020), which involved in protein mobilization during seed germination (Bewley et al., 2013). TIP1 and TIP2 are preferentially localized in the lytic vacuoles formed by PSV and vegetative storage proteins vacuoles, respectively (Maurel et al., 2008). It has also been observed that ZmTIP1 was highly expressed in dividing and expanding cells of seedling roots (Barrieu et al., 1998; Chaumont et al., 1998). Here, we further clarified the expression trend of ZmTIP1s, i.e., that they appeared in only the protruding radicle, increased gradually with the germination process, and increased to substantial levels in the roots of seedlings (Figure 3B). Numerous studies confirmed the spatiotemporal expression of TIP aquaporins. However, the mechanisms governing the shift of TIPs remain unclear. The study also observed the disappearance of ZmTIP3-1 after radicle emergence was only at transcription levels, not at the protein level (Figures 5C,D), suggesting that ZmTIP3-1 was still functioning somehow. Considering the high accumulation of *ZmTIP3-1* in mature kernels and dry seeds (Supplementary Figures 7, 8), we further speculated that the transcription of *ZmTIP3-1* did not occur while the stored *ZmTIP3-1* mRNA in dry seed did work during seed germination. *Arabidopsis* AtTIP1-1 and AtTIP3-1 were both found in the central vacuole of seedlings (Gattolin et al., 2011). In barley seed development, HvTIP3-1 interacted with HvTIP1-2 to form a heterotetramer, which facilitates the water transport capacity (Utsugi et al., 2015). In maize germination, further investigation is required to determine whether there is a physical interaction between ZmTIP3 and ZmTIP1 or TIP2 to supply considerable water for rapid seedling growth.

Seed germination and seedling emergence, beginning from a quiescent state to a metabolically active state in a short period, involve a series of physiological, biochemical, and morphological changes, and various tissues or organs such as the radicle, coleoptile, plumule, and mesocotyl in cereal seeds (Bewley et al., 2013). These activities cannot be separated from AQP-facilitated water uptake and transport in seed and seedlings (Obroucheva and Sin’kevich, 2010; Obroucheva, 2013; Obroucheva et al., 2017). AQPs exhibited exquisite spatiotemporal-specific expression patterns in the germination process, which is complementary to the versatile roles of AQPs in response to the establishment of new tissues/organs and various signals such as hormones or environmental stresses (Maurel et al., 2008; Li et al., 2014; Kapilan et al., 2018). Therefore, we suggest that the germinating seed may be an excellent model to examine the regulation mechanisms of the shift of the different AQPs during these sequential developmental stages.

### Regulation of Maize Aquaporins

The finely tuned biological functions of plant AQPs can be regulated in multiple ways, including transcriptional and posttranslational modifications (Maurel et al., 2008, 2015; Chaumont and Tyerman, 2014). The transcriptional response to various signals and stimuli was mainly mediated by *cis*-acting regulatory elements. A bioinformatic promoter *cis*-element analysis of ZmAQP was performed to understand its versatile roles and dynamic changes during different development stages. The *cis*-elements of ZmAQP promoters were identified and classified into four major groups: stress, phytohormone, and light response, as well as plant growth-related *cis*-elements (Supplementary Figure 11).

Here, the results indicated that most ZmAQPs contain rich *cis*-elements in response to phytohormones and light. ABRE, as a major *cis*-acting element in ABA-responsive gene expression, and G-box (involved in light response) were the most abundant *cis*-acting elements (Supplementary Figures 11B–D). The ABA-signaling pathway is central to stress-responses in plants (Zhu, 2016). At the same time, we found that most of the ZmAQP genes had multiple stress-responsive elements, such as ARE (essential for the anaerobic induction), LTR (involved in low-temperature stress), and MBS (MYB binding site involved in drought-stress) (Supplementary Figure 11A). Numerous studies have implicated plant AQPs in abiotic stress regulation (Vera-Estrella et al., 2004; Xu et al., 2013; Ge et al., 2014; Zhang D. et al., 2015; Rios et al., 2017; Footitt et al., 2019). ABA has an essential role in seed development and germination. For seed-specific ZmTIP3s, an abundance of ABRE elements was identified (Supplementary Figure 11A), suggesting a potential role of ZmTIPs in ABA responses. The ABA content gradually increased as the grain developed, and the ZmTIP3 expression also exhibited a steadily rising trend (Supplementary Figure 7). Studies of *Arabidopsis* and barley have shown that the transcripts of TIP3s are regulated by GA and ABA during germination (Lee et al., 2015; Footitt et al., 2019). Furthermore, the transcription factor ABSCISIC ACID INSENSITIVE 3 (ABI3) directly binds to the RY motif of AtTIP3 promoters, which in turn induces AtTIP3 expression (Mao and Sun, 2015). However, the seed-specific RY-motif was found only in ZmTIP3-2, not in ZmTIP3-1 (Supplementary Figure 11C). Our data are in agreement with the findings of Footitt et al. (2019) for the different responses of TIP3-1 and TIP3-2 to hormones (Supplementary Figure 9A), which indicates they perform different functions, despite that their sequences shared high similarity. Light-responsive G-box element was the most enriched *cis*-acting element in the promoters of ZmAQP (Supplementary Figure 11D). PIF3 (Phytochrome interacting factor 3) and HY5 (Elongated hypocotyl 5), as two critical transcription factors in the light signaling pathway, can bind to G-box elements of light-responsive genes to regulate seedling morphogenesis (Liu et al., 2013; Zhao et al., 2019). Promoter *cis*-element analysis showed *ZmTIP1s* and *ZmTIP2s* contained abundant G-box elements (Supplementary Figure 11), which was in line with the extremely high expression of *ZmTIPs* in the mesocotyl of 4-d-old seedlings (Figure 3B). Additionally, ethylene, facilitating seedling emergence through the soil (Zhong et al., 2014), significantly induced the expression of *ZmTIP1-1* (Supplementary Figure 9C). These results suggest that ZmTIPs may contribute to seedling establishment. The pivotal role of gibberellin (GA) in seed germination is self-evident. ZmPIP genes contain a relatively large number of GA-responsive elements, including P-box, GARE-box, and TATC-box (Supplementary Figure 11B). On the contrary, ZmTIP1, TIP2, and TIP3 contain few GA-related elements, except for ZmTIP1-2 (Supplementary Figure 11), and its expression was markedly increased under GA treatment during seedling emergence (Supplementary Figure 9C). How the environmental signal-related *trans*-acting factors (or transcription factors) recognize and interact with the *cis*-acting elements, thereby positively or negatively regulating ZmAQPs at the transcriptional level, should be further investigated.

The aquaporin activity, trafficking, and interaction with other partners are influenced by post-translational modifications (Chaumont and Tyerman, 2017). Phosphorylation is a major posttranslational modification of aquaporins. Hence, we performed the prediction of maize aquaporin phosphorylation sites. According to the prediction results, serine was the most dominant phosphorylated residue in ZmAQPs, and the sites were relatively conserved within each subfamily (Supplementary Figure 1). The results showed that ZmPIPs had N-terminal Ser- and Thr-phosphorylated residues. In addition, ZmPIP2s had highly conserved Ser-phosphorylated sites in the C-terminal (Supplementary Figure 1). The identified sites in the previous study are annotated. These results prove the same in accordance with the previous analysis based on mass spectrometry and phosphoproteomic analysis (Van Wilder et al., 2008; Hu X. et al., 2015), thus confirming the prediction analysis. In ZmTIPs, the most highly conserved phosphorylation site was the Ser-residue located in the α-helixes of HE (Supplementary Figure 1). However, the site was not present in seed-specific ZmTIP3s. Calcium-dependent protein kinases acting in aquaporin phosphorylation have been identified (Harvengt et al., 2000; Sjövall-Larsen et al., 2006). Phosphorylation of AQPs confers plants the ability to gate aquaporins, which strongly involved in water channel activity (Chaumont and Tyerman, 2017; Daniels and Yeager, 2019). Moreover, the identification of interaction partners of AQPs may facilitate the discovery of novel protein kinases for phosphorylating AQPs, as well as new heterotetramers. There were studies indicating that plants had the capacity to regulate water transport activity and subcellular localization through heterotetramers (Zelazny et al., 2007; Utsugi et al., 2015; Bellati et al., 2016; Berny et al., 2016). The AQP protein-interacting networks were constructed based on the STRING. We identified a total of 104 ZmAQP-interacting proteins, mainly involved in sugar transport, transcription factors, and protein kinases (Supplementary Figure 10). The detailed information of the interacting proteins is shown in Supplementary Table 4. For example, the protein kinases will help to provide a better understanding of the phosphorylation of ZmAQPs. More rigorous experimental approaches are required to validate these predictive results. Further identification and characterization of *cis*-regulatory elements and posttranslational modifications may help to reveal the regulation mechanism of plant AQPs.

### ZmTIPs: Are They Markers of Seed and Seedling Vigor?

Seed vigor, a comprehensive trait for evaluating seed quality, can be demonstrated by germination characteristics, such as the rate and uniformity of seed germination and seedling growth (ISTA, 2014). First, our study clarified the shift in ZmTIP3s and TIP1s/2s at the transcriptional level during seed germination (Figure 3B and Supplementary Figure 5B). Decreased the expression of TIP3s was relative to the coalescence of PSVs to central vacuoles (Novikova et al., 2014; Lee et al., 2015). Generally, TIP3s and TIP1s are considered to be characteristic of PSVs and vegetative vacuoles (Maurel et al., 2008). Early studies have proposed hypotheses about the roles of TIP3 in preparation for germination (Maurel, 1997). Aquaporins ZmTIP3s could be biomarkers of seed vigor based on proteomics analysis in our research for the first time (Li et al., 2018). The study demonstrated that a peak in ZmTIP3s expression was observed at maturity and decreased during germination (Figure 3C and Supplementary Figure 7). In parallel, we confirmed that ZmTIP3-1 improves the seed protein content and enhances seedling growth (Figures 5, 6). Because of the evaluation of seed vigor by the protein content of seed in cereals (Wen et al., 2018), we thus consider whether the ZmTIP3 expression levels can be used as a marker of the protein content in maize. Unfortunately, no significant positive correlation between the ZmTIP3 transcript accumulation and the stored protein content of seeds in about 270 maize accessions was observed, which may be attributed to the differences in the genetic background of the materials used. The finding is in alignment with previous experiments on soybean (Melroy and Herman, 1991). We suggest that ZmTIP3-1 might promote stored protein mobilization and thereby increase seedling growth. After radicle emergence, ZmTIP3s were replaced with ZmTIP1s/2s to perform functions in the tonoplast. Given the importance of mesocotyl elongation in seedling establishment and the extremely high expression of ZmTIP1s in the mesocotyl, A more detailed analysis is needed to elucidate whether and how ZmTIPs regulate seedlings growing out of the soil. Further, is the high accumulation of ZmTIP3 related to water uptake during seed imbibition? How does the dual localization of ZmTIP3 occur? Does ZmTIP3-2 have a different function in regulating seed germination? Answers to the above questions of both fundamental and practical importance remain to be revealed.

## Conclusion

In conclusion, based on the latest maize genome database, we identified 41 ZmAQPs and performed a prediction analysis of their basic information involved in the physicochemical characteristics and structural properties. We focused on the expression profiles of ZmAQPs during the seed germination and seedling emergence, and further confirmed the positive roles of seed-specific ZmTIP3-1 in early seedling growth. The results provide direct genetic evidence of the functions of ZmAQPs in seed germination.

## Data Availability Statement

The original contributions presented in the study are included in the article/Supplementary Material, further inquiries can be directed to the corresponding author/s.

## Author Contributions

LZ and CZ conceived the research. YS, ZL, and JS performed most of the experiments. CZ, LZ, and YS supervised the experiments and analyzed the data. CW and YL provided technical assistance and suggestions for writing. LZ and YS wrote the manuscript. All authors commented on the manuscript.

## Conflict of Interest

The authors declare that the research was conducted in the absence of any commercial or financial relationships that could be construed as a potential conflict of interest.

## Publisher’s Note

All claims expressed in this article are solely those of the authors and do not necessarily represent those of their affiliated organizations, or those of the publisher, the editors and the reviewers. Any product that may be evaluated in this article, or claim that may be made by its manufacturer, is not guaranteed or endorsed by the publisher.

## Footnotes

1
http://pfam.xfam.org/

2
http://www.uniprot.org/

3
https://www.ncbi.nlm.nih.gov/assembly/GCF_902167145.1

4
https://web.expasy.org/protparam/

5
https://wolfpsort.hgc.jp/

6
http://www.cbs.dtu.dk/services/TMHMM/

7
https://services.healthtech.dtu.dk/service.php?NetPhos-3.1

8
http://www.sbg.bio.ic.ac.uk/phyre2/html/page.cgi?id=index

9
https://string-db.org/
