# Supplementary material for: Genome-Wide Identification of Maize Aquaporin and Functional Analysis During Seed Germination and Seedling Establishment
Source: Front Plant Sci. 2022 Jan 27;13:831916. doi: 10.3389/fpls.2022.831916 (PMC8828918; doi:10.3389/fpls.2022.831916)
Supplement: Supplementary file 1 [file Data_Sheet_1.PDF]

# Supporting Figures

```
AtTIP2-1   1   ------MSHHHHHHHHHHDSNGIPTENLYFQGAG--------------------------   28
SoPIP2-1   1   ------MSKEVS-----------EEAQAHQHGKD--------YVDPPP------------   23
ZmPIP1-1   1   -----MEGKEEDVRLGANKFSERQPIGTAAQGT----DDKD-YKEPPP------------   38
ZmPIP1-2   1   -----MEGKEEDVRLGANKFSERQPIGTAAQGAA---DDKD-YKEPPP------------   39
ZmPIP1-3   1   -----MEGKEEDVRLGANKFSERQPIGTAAQGAGAGDDDKD-YKEPPP------------   42
ZmPIP1-5   1   -----MEGKEEDVRLGANRYSERQPIGTAAQGT----EEKD-YKEPPP------------   38
ZmPIP2-1   1   ------MGKDD------------VIESGAGGGEF---AAKD-YTDPPP------------   26
ZmPIP2-2   1   ------MGKDD------------VVQSGAGGGEF---AAKD-YTDPPP------------   26
ZmPIP2-3   1   ------MAKQD------------IEASGPEAGEF---SAKD-YTDPPP------------   26
ZmPIP2-4   1   ------MAKD-------------IEASGPEAGEF---SAKD-YTDPPP------------   25
ZmPIP2-5   1   ------MAKD-------------IEAAAAHEGKD--------YSDPPP------------   21
ZmPIP2-6   1   ------MGKE-------------VDVSTLEAGGV---RDRD-YADPPP------------   25
ZmPIP2-9   1   ------MGK-E------------VDVSTLEAGGV-----RD-YADPPP------------   23
ZmPIP2-8   1   ------MDTCH------------QSIETADGKKD--------YSDPVP------------   22
ZmPIP2-7   1   ------MSKGD------------VVIETAAEAEA---TAKAPYWDPPP------------   27
ZmTIP1-1   1   -----------------------MPINR--------------------------------   5
ZmTIP1-2   1   -----------------------MPVSRIAV-----------------------------   8
ZmTIP2-1   1   ------MVK---------------------------------------------------   3
ZmTIP2-2   1   ------MVK---------------------------------------------------   3
ZmTIP2-3   1   ------MVK---------------------------------------------------   3
ZmTIP2-4   1   ------------------------------------------MVKP--------------   4
ZmTIP3-1   1   ------------------------MSTGVRPGRR--------------------------   10
ZmTIP3-3   1   MNMIRAVRRR--------------------------------------------------   10
ZmTIP3-2   1   ------MST----------------ATGVRAGRR--------------------------   12
ZmTIP3-4   1   ----MLPGRHPARR----------------------------------------------   10
ZmTIP4-1   1   ------MAKLMNKLVDS-------------------------------------------   11
ZmTIP4-2   1   ------MSHSPLPPPVFQNISLRFSESFSLQGTETTGAFTPPAFPSPPGTTGLLAIIRPS   54
ZmTIP4-3   1   ------MGK---------------------------------------------------   3
ZmTIP4-4   1   ------MAK---------------------------------------------------   3
ZmTIP5-1   1   ------MASN--------------------------------------------------   4
ZmNIP1-1   1   -----MAGGGDHS----------QTNGGHVDQRALEEGRKEEFADQ--------------   31
ZmNIP1-3   1   -----MAGAEVANGVHEGALDLEEGRGGGVDGAGCESSEQDG------------------   37
ZmNIP1-4   1   -----MARREDDSYTNASVFETSVEDGRKDKSES-------YAVDEPP------------   36
ZmNIP2-1   1   ------MSTNSRSNSRANFNNEIHDIGTAQNSSM-----------PPT------------   31
ZmNIP2-4   1   ------MSTNSRANSRANFNNEIHDIGTAVHNS---------SSLPPA------------   33
ZmNIP2-2   1   ---MAAASTTSRTNSRVNYSNEIHDLSTVQSGSVVPTLF---------------------   36
ZmNIP2-3   1   ----MAASTTSRTNSRVNYSNEIHDLSTVQGGSAAAAAAALFY--PDS------------   42
ZmNIP3-1   1   -----MEPGSTPPNGSAPATPGTPAPLFSSGGPRVDSLSYERKSMPRCKCLPLPAVEGWG   55
ZmNIP3-2   1   MAEHATTGVEEQRQVAISMCSAPTPSKLVAVESS--SLQKLMLKSPPQA-----------   47
ZmNIP4-1   1   MAAMMMDSTAEK---------DLAGDGAAVSGHGQDLERSCHDQEPAA------------   39
ZmSIP1-1   1   ------------------------------------------------------------   0
ZmSIP1-2   1   ------------------------------------------------------------   0
ZmSIP2-1   1   ------M-----------------------------------------------------   1
```

**TM1**

| | | | |
|---|---|---|---|
| AtTIP2-1 | 29 | -VAF-------GSFDDSFSLA--SLRAYLAEFISTLLFVFAG-VGSAIAYAKLTSD---- | 73 |
| SoPIP2-1 | 24 | -APF-------FDLGELKLWS--FWRAAIAEFIATLLFLYIT-VATVIGHSKET------ | 66 |
| ZmPIP1-1 | 39 | -APL-------FEPGELKSWS--FYRAGIAEFVATFLFLYIS-ILTVMGVSKST------ | 81 |
| ZmPIP1-2 | 40 | -APL-------FEPGELKSWS--FYRAGIAEFVATFLFLYIT-ILTVMGVSKST------ | 82 |
| ZmPIP1-3 | 43 | -APL-------FEPGELKSWS--FYRAGIAEFVATFLFLYIT-VLTVMGVSKST------ | 85 |
| ZmPIP1-5 | 39 | -APL-------FEAEELTSWS--FYRAGIAEFVATFLFLYIS-ILTVMGVSKSS------ | 81 |
| ZmPIP2-1 | 27 | -APL-------IDAAELGSWS--LYRAVIAEFIATLLFLYIT-VATVIGYKHQTDASAS- | 74 |
| ZmPIP2-2 | 27 | -APL-------VDAAELGSWS--LYRAVIAEFIATLLFLYVT-VATVIGYKHQTDASASG | 75 |
| ZmPIP2-3 | 27 | -APL-------IDADELTKWS--LYRAVIAEFIATLLFLYIT-VATVIGYKHQTDAAAS- | 74 |
| ZmPIP2-4 | 26 | -APL-------IDAEELTQWS--LYRAVIAEFIATLLFLYIT-VATVIGYKHQTDASAS- | 73 |
| ZmPIP2-5 | 22 | -APL-------VDAEELTKWS--LYRAVIAEFVATLLFLYIT-VATVIGYKHQTDAAAS- | 69 |
| ZmPIP2-6 | 26 | -APL-------IDIDELGKWS--LYRAVIAEFVATLLFLYIT-VATVIGYKHQTDASAS- | 73 |
| ZmPIP2-9 | 24 | -APL-------IDVDELGKWS--LYRAVIAEFVATLLFLYIT-VATVIGYKHQTDASAS- | 71 |
| ZmPIP2-8 | 23 | -APF-------VNAGELGKWS--LYRAVIAEFVATLLFVYVT-LATVIGHKREAES---- | 67 |
| ZmPIP2-7 | 28 | -APL-------LDTSELKKWS--LYRALIAEFMATLIFLYVS-VATVIGYKSQSQA---- | 72 |
| ZmTIP1-1 | 6 | -IAL-------GSHQEVYHPG--ALKAAFAEFISTLIFVFAG-QGSGMAFSKLTGG---- | 50 |
| ZmTIP1-2 | 9 | -----------GAPGELSHPD--TAKAAVAEFISTLIFVFAG-SGSGMAFSKLTDG---- | 50 |
| ZmTIP2-1 | 4 | -LAF-------GSVGDSFSAT--SIKAYVAEFIATLLFVFAG-VGSAIAYGQLTNG---- | 48 |
| ZmTIP2-2 | 4 | -LAF-------GSVGDSFSVT--SIKAYVAEFIATLLFVFAG-VGSAIAFGQLTNG---- | 48 |
| ZmTIP2-3 | 4 | -LAF-------GSFRDSLSAA--SLKAYVAEFIATLLFVFAG-VGSAIAYSQLTKG---- | 48 |
| ZmTIP2-4 | 5 | --AF-------GSFGDSFSAA--SLKAYAAEFIATLLFVFAG-VGSAIAYSQLTKG---- | 48 |
| ZmTIP3-1 | 11 | -FTV-------GRSEDATHPD--TIRAAISEFIATAIFVFAA-EGSVLSLGKMYH----- | 54 |
| ZmTIP3-3 | 11 | -FTV-------GHLATAKDPA--TLRHAAAELLATAIFVFAA-EGATLSLGRMHH----- | 54 |
| ZmTIP3-2 | 13 | -FTV-------GRSEDATHPD--TIRAAISEFIATAIFVFAA-EGSVLSLGKMYHDHS-- | 59 |
| ZmTIP3-4 | 11 | -----------ADTTGTGPLLPDATRAVVSEFVATAMFVFAA-EGSVYGLWKLYK----- | 53 |
| ZmTIP4-1 | 12 | -----------FEHDEILDVG--CVRAVLAELVLTFLFVFTG-VSAAMAAGSDGKPG--- | 54 |
| ZmTIP4-2 | 55 | MAKLVNKLVDSFDHHEAPAPDVGCVRAVLAELVLTFLFVFTG-VSASMAAGAGGKPG--- | 110 |
| ZmTIP4-3 | 4 | -LTL-------GHRGEASEPD--FFRGVLGELVLTFLFVFIG-VGAAMTDGATTK----- | 47 |
| ZmTIP4-4 | 4 | -FAL-------GHHREASDAG--CVRAVLAELILTFLFVFAG-VGSAMATGKLAGG---- | 48 |
| ZmTIP5-1 | 5 | -NLL-------VDLKRCFSAP--SLRSYLAEFISTFLFVFTA-VGSAISARMLTTPDVT- | 52 |
| ZmNIP1-1 | 32 | -----------GCAAMVVSVP--FIQKIIAEIFGTYFLMFAG-CGAVTINA--------- | 68 |
| ZmNIP1-3 | 38 | -----------AGRRPMFSVP--FVQKILAEALGTYFLIFAG-CAAVAVNL--------- | 74 |
| ZmNIP1-4 | 37 | -QPV-------DDALCGMSTSVSFIQQLIAEFLATFFLIFAG-CGVIAVN---------- | 77 |
| ZmNIP2-1 | 32 | -YYD-------RSLADIFPPH--LLKKVVSEVVSTFLLVFVT-CGAAGIYG--------- | 71 |
| ZmNIP2-4 | 34 | -YYD-------RSLADMFPPH--LLKKVVSEVVSTFLLVFVT-CGAAGIYG--------- | 73 |
| ZmNIP2-2 | 37 | -YPD-------KSIADIFPPH--LGKKVISEVVATFLLVFVT-CGAASIYG--------- | 76 |
| ZmNIP2-3 | 43 | -----------KSIADIFPPH--LGKKVISEVVATFLLVFVT-CGAASIYG--------- | 79 |
| ZmNIP3-1 | 56 | VATH-------TCVVEIPAPDVSLTRKLGAEFVGTFILIFFA-TAAPIVNQ--------- | 98 |
| ZmNIP3-2 | 48 | -DAH-------GDEQQGREVP--LAKKVAAEFVGTFILMFAV-VSTVVADA--------- | 87 |
| ZmNIP4-1 | 40 | -ADG-------ASSRGLAIGR--FVRELMVEGVASFLLVFWSAVAALMQ----------- | 78 |
| ZmSIP1-1 | 1 | -----------MAMGA-------TVRAAAADAVVTFLWVL---CASALGASTAAVTSYL- | 38 |
| ZmSIP1-2 | 1 | -----------MAMGE-------ALRAAAADAVVTFLWVL---CVSTLGASTT------- | 32 |
| ZmSIP2-1 | 2 | -SPA-------PSRPRIRPWL------VVGDLALAAAWVCAGALVKLLVYGGL------- | 40 |

```
                                         TM2                 HB
AtTIP2-1   74   ----AALDTPGLVAIAVC-------HGFA-LFVAVAIGANISGGHVNPAVTFGLAVGGQ-   120
SoPIP2-1   67   ----VVCGSVGLLGIAWA-------FGGM-IFVLVYCTAGISGGHINPAVTFGLFLARK-   113
ZmPIP1-1   82   ----SKCATVGIQGIAWS-------FGGM-IFALVYCTAGISGGHINPAVTFGLFLARK-   128
ZmPIP1-2   83   ----SKCATVGIQGIAWS-------FGGM-IFALVYCTAGISGGHINPAVTFGLFLARK-   129
ZmPIP1-3   86   ----SKCATVGIQGIAWS-------FGGM-IFALVYCTAGISGGHINPAVTFGLFLARK-   132
ZmPIP1-5   82   ----SKCATVGIQGIAWS-------FGGM-IFALVYCTAGISGGHINPAVTFGLFLARK-   128
ZmPIP2-1   75   -GADAACGGVGVLGIAWA-------FGGM-IFVLVYCTAGISGGHINPAVTFGLFLARK-   124
ZmPIP2-2   76   AGADAACGGVGVLGIAWA-------FGGM-IFVLVYCTAGISGGHINPAVTFGLFLARK-   126
ZmPIP2-3   75   -GPDAACGGVGILGIAWA-------FGGM-IFILVYCTAGISGGHINPAVTFGLFLARK-   124
ZmPIP2-4   74   -GPDAACGGVGILGIAWA-------FGGM-IFILVYCTAGISGGHINPAVTFGLFLARK-   123
ZmPIP2-5   70   -GPDAACGGVGVLGIAWA-------FGGM-IFILVYCTAGVSGGHINPAVTFGLFLARK-   119
ZmPIP2-6   74   -GPDAACSGVGILGIAWA-------FGGM-IFILVYCTAGISGGHINPAVTFGLFLARK-   123
ZmPIP2-9   72   -GPGAACGGVGVLGIAWA-------FGGM-IFILVYCTAGISGGHINPAVTFGLFLARK-   121
ZmPIP2-8   68   ----QPCGSVGVLGIAWS-------FGGM-IFVLVYCIAGISGGHINPAVTFGLLLARK-   114
ZmPIP2-7   73   ----EACTGVGFLGVAWS-------FGAT-IFILVYCTGGISGGHINPAVTFGLFVGRK-   119
ZmTIP1-1   51   ----GPTTPAGLIAAAVA-------HAFA-LFVAVSVGANISGGHVNPAVTFGAFVGGN-   97
ZmTIP1-2   51   ----GAATPAGLIAASLA-------HALA-LFVAVSVGANISGGHVNPAVTFGAFVGGN-   97
ZmTIP2-1   49   ----GALDPAGLVAIAIA-------HALA-LFVGVSVAANISGGHLNPAVTFGLAVGGH-   95
ZmTIP2-2   49   ----GALDPAGLVAIAVA-------HALA-LFVGVSVAANTSGGHLNPAVTFGLAVGGH-   95
ZmTIP2-3   49   ----GALDPAGLVAIAIA-------HAFA-LFVGVSMAANISGGHLNPAVTFGLAVGGH-   95
ZmTIP2-4   49   ----GALDPAGLVAIAIA-------HAFA-LFVGVSMAANISGGHLNPAVTFGLAVGGH-   95
ZmTIP3-1   55   ----DMSTAGGLVAVALA-------HALA-LAVAVAVAVNISGGHVNPAVTFGALVGGR-   101
ZmTIP3-3   55   ----HDKGGGGLVAVALA-------HALA-LAAAVGCAANISGGHVNPAVTFGALLAGR-   101
ZmTIP3-2   60   ----TISTAGGLVAVALA-------HALG-LAVAVAVAVNVSGGHVNPAVTFGALVGGR-   106
ZmTIP3-4   54   ----DTATPGGLLAVAIA-------HTLA-LVAAVAVASNASGGHVNPAVTFGLLVGRR-   100
ZmTIP4-1   55   ----DAMPMATLAAVAIA-------HALA-AGVLVTAGFHVSGGHLNPAVTVGLMVRGH-   101
ZmTIP4-2   111  ----EAMPMATLAAVAIA-------HALA-AGVLVTAGFHVSGGHLNPAVTVGILVRGH-   157
ZmTIP4-3   48   ----GSTAGGDLTAVALG-------QALV-VAVIATAGFHISGGHVNPAVTLSLAVGGH-   94
ZmTIP4-4   49   ----GGDTVVGLTAVALA-------HTLV-VAVMVSAGLHVSGGHINPAVTLGLAATGR-   95
ZmTIP5-1   53   ----SSAGPLVATAVAQA-------FG---LFAAVLIAADVSGGHVNPAVTFAYAIGGR-   97
ZmNIP1-1   69   ----SKNGQITFPGVAIV-------WGLA-VMVMVYAVGHISGAHFNPAVTLAFATSGR-   115
ZmNIP1-3   75   ----RTGGTVTFPGICAV-------WGLA-VMVMVYSVGHISGAHLNPAVSLAFATCGR-   121
ZmNIP1-4   78   ----DKNGMATFPGIAVV-------WGMV-VMAMIYAVGHVSGAHINPAVSVGFAISGR-   124
ZmNIP2-1   72   ----SDKDRISQLGQSVA-------GGLI-VTVMIYAVGHISGAHMNPAVTLAFAVFRH-   118
ZmNIP2-4   74   ----SDKDRISQLGQSVA-------GGLI-VTVMIYAVGHISGAHMNPAVTLAFAVFRH-   120
ZmNIP2-2   77   ----EDNRRISQLGQSVA-------GGLI-VTVMIYATGHISGAHMNPAVTLSFACFRH-   123
ZmNIP2-3   80   ----EDNARISQLGQSVA-------GGLI-VTVMIYATGHISGAHMNPAVTLSFACFRH-   126
ZmNIP3-1   99   ----KYGGAISPFGNAAC-------AGLA-VATVILSTGHISGAHLNPSLTIAFAALRH-   145
ZmNIP3-2   88   ----QHGGAEGLVGVAAA-------AGLA-VVAVVLAVVSVSGSHLNPAVSLAMGVFGY-   134
ZmNIP4-1   79   ----EMHGTLTFPMVCLV-------VALTVVFVLCWLG----PAHFNPAVTVTFTVFGY-   122
ZmSIP1-1   39   ----GVQEGAGHYALLVT----TSLLSVL-LFTFDLLCGALGGASFNPTDFAASYAAGL-   88
ZmSIP1-2   33   ----AVTSYLRLQGVHFALLVTVSLLSVL-LFVFNILCDALGGASFNPTGVAAFYAAGV-   86
ZmSIP2-1   41   ----GLGGRPEAEAVKVS-------LSLVYMFLFAWLEAASGGASYNPLTVLAAALASHG    89
```

TM3

| | | | |
|---|---|---|---|
| AtTIP2-1 | 121 | ---IT--VITGVFYWIAQLLGSTAACFLLKYVTGG-----LAVPTHSVAAGLGSIEGVVM | 170 |
| SoPIP2-1 | 114 | ---VS--LLRALVYMIAQCLGAICGVGLVKAFMKG-PYNQFGGGANSVALGYNKGTALGA | 167 |
| ZmPIP1-1 | 129 | ---LS--LTRAVFYIIMQCLGAICGAGVVKGFQQG-LYMGNGGGANVVAPGYTKGDGLGA | 182 |
| ZmPIP1-2 | 130 | ---LS--LTRALFYIIMQCLGAVCGAGVVKGFQQG-LYMGNGGGANVVAPGYTKGDGLGA | 183 |
| ZmPIP1-3 | 133 | ---LS--LTRAIFYIIMQCLGAICGAGVVKGFQQG-LYMGNGGGANVVAPGYTKGDGLGA | 186 |
| ZmPIP1-5 | 129 | ---LS--LTRALFYMVMQCLGAICGAGVVKGFQEG-LYMGAGGGANAVNPGYTKGDGLGA | 182 |
| ZmPIP2-1 | 125 | ---VS--LVRALLYIVAQCLGAICGVGLVKAFQSA-YFDRYGGGANSLASGYSRGTGLGA | 178 |
| ZmPIP2-2 | 127 | ---VS--LVRALLYMVAQCLGAVCGVGLVKAFQSA-YFDRYGGGANSLASGYSRGAGLGA | 180 |
| ZmPIP2-3 | 125 | ---VS--LVRALLYIIAQCLGAICGVGLVKGFQSA-YYVRYGGGANELSDGYSKGTGLAA | 178 |
| ZmPIP2-4 | 124 | ---VS--LVRALLYIIAQCLGAICGVGLVKGFQSA-YYVRYGGGANELSDGYSKGTGLAA | 177 |
| ZmPIP2-5 | 120 | ---VS--LVRALLYIVAQCLGAICGVGLVKGFQSA-FYVRYGGGANELSAGYSKGTGLAA | 173 |
| ZmPIP2-6 | 124 | ---VS--LVRALLYMAAQSLGAICGVALVKGFQSG-FYARYGGGANEVSAGYSTGTGLAA | 177 |
| ZmPIP2-9 | 122 | ---VS--LVRALLYMAAQSLGAICGVALVKGFQSG-LYARYGGGANEVSAGYSTGTELAA | 175 |
| ZmPIP2-8 | 115 | ---LS--LVRAALYVVAQCLGAMCGAGLVKAFHGAHWYLRYGGGANELAAGYSKGAGLGA | 169 |
| ZmPIP2-7 | 120 | ---LS--LVRTLLYIAAQCLGAVCGVGIVKAIMKH-PYNSLGGGANEVATGYSVGGALAA | 173 |
| ZmTIP1-1 | 98 | ---IT--LFRGLLYWVAQLLGSTVACFLLRFSTGG-----QATGTFGLT-GVSVWEALVL | 146 |
| ZmTIP1-2 | 98 | ---IS--LLKALVYWVAQLLGSVVACLLLKIATGG-----AALGAFSLSAGVGAMNAVVL | 147 |
| ZmTIP2-1 | 96 | ---IT--ILTGVFYWVAQLLGATVACLLLGFVTHG-----KAIPTHAVA-GISELEGVVF | 144 |
| ZmTIP2-2 | 96 | ---IT--VLTGLFYWVAQLLGASVACLLLRFVTHG-----KAIPTHGVSGGTTELEGVVF | 145 |
| ZmTIP2-3 | 96 | ---IT--ILTGILYWVAQLLGASVACFLLQYVTHG-----QAIPTHGVS-GISEIEGVVM | 144 |
| ZmTIP2-4 | 96 | ---IT--ILTGVFYWVAQLLGASVACLLLQFVTHG-----QAIPTHGVS-GISEIEGVVM | 144 |
| ZmTIP3-1 | 102 | ---VS--LVRAVLYWVAQLLGAVAATLLLRLATGG-----MRPPGFALASGVGDWHAVLL | 151 |
| ZmTIP3-3 | 102 | ---IC--LVRSLVYWAAQLLGAVAAALVLRLATGG-----MHLPEYALAGGVSGWNAAVL | 151 |
| ZmTIP3-2 | 107 | ---VS--LVRAVLYWAAQLLGAVAATLLLRLATGG-----ARPPGFALASGVGDGHAVLL | 156 |
| ZmTIP3-4 | 101 | ---IS--FGRAAVYWLAQMLGAVVASLLLTLVSGG-----TRPVGFGLVRGVHERHALLL | 150 |
| ZmTIP4-1 | 102 | ---IT--KLRAVLYVAAQLLASSAACVLLRFLSGG-----MVTPVHALGRGISPMQGLVM | 151 |
| ZmTIP4-2 | 158 | ---IT--KLRALLYVAAQLLASSLACILLRYLSGG-----MVTPVHALGAGISPMQGLVM | 207 |
| ZmTIP4-3 | 95 | ---VT--LFRSSLYIAAQMLGSSAACFLLRWLTGG-----LATPVHALAEGVGALQGVVA | 144 |
| ZmTIP4-4 | 96 | ---IT--LFRSALYVAAQLLGSTLACLLLAFLAVA----DSGVPVHALGAGVGALRGVLM | 146 |
| ZmTIP5-1 | 98 | ---IG--VPSAMFYWASQLLGATFACLSLNLFSAG-----EEVPTTRIAVAMTGFGGAVL | 147 |
| ZmNIP1-1 | 116 | ---FP--WRQLPAYVLAQMLGATLASGTLRLMFGG-RHEHFPG---TLPTG-SEVQSLVI | 165 |
| ZmNIP1-3 | 122 | ---FP--WRQVPAYAAAQVTGATAASLTLRLLFGSAREHFFG----TVPAG-SDAQSLVV | 171 |
| ZmNIP1-4 | 125 | ---FP--WRKVPAYMLVQTVAATMASLVLRLMFGR---QHELASVTVPAPGGSIFQSLVL | 176 |
| ZmNIP2-1 | 119 | ---FP--WIQVPFYWAAQFTGGSICASFVLKAVLHP-----IAVLGTTTPTG-PHWHSLVI | 167 |
| ZmNIP2-4 | 121 | ---FP--WIQVPFYWAAQFTGAICASFVLKAVLHP-----IAVLGTTTPAG-PHWHSLII | 169 |
| ZmNIP2-2 | 124 | ---FP--WIQVPFYWAAQFTGAMCAAFVLKAVLHP-----IAVIGTTTPSG-PHWHALLI | 172 |
| ZmNIP2-3 | 127 | ---FP--WIQVPFYWAAQFTGAMCAAFVLKAVLQP-----IAVIGTTTPSG-PHWHALAI | 175 |
| ZmNIP3-1 | 146 | ---FP--WLQVPAYVAVQALASVCAAFALKGVFHP----FLSGGVTVPDATVSTAQAFFT | 196 |
| ZmNIP3-2 | 135 | ---LP--RAHVLPYAAAQTAGSAAAAFLAKAMYRP-----ADPAVMATVPRVGAAQAFFL | 184 |
| ZmNIP4-1 | 123 | ---LS--WTKLPFYVAAQLAGSLLACLSANGVMEPRAEHFYG---TVPMAGGDTRLPFLL | 174 |
| ZmSIP1-1 | 89 | ---DSPSLFSVALRFPAQAAGAVGGALAISELMPAQYKHTLAGPSLKVDP----HTGALA | 141 |
| ZmSIP1-2 | 87 | ---TSPSLFSIALRLPAQAAGAVGGALAISELMPAQYRHMLGGPSLKVDP----HTGAGA | 139 |
| ZmSIP2-1 | 90 | GPAVY--LFTAFARIPAQVIGAVLGVKLIQVTFPN------VGKGARLSVG--AHHGALA | 139 |

TM4 TM5

| | | | |
|---|---|---|---|
| AtTIP2-1 | 171 | EIIITFALVYTVYATAADP-K---KG------SLGTIAPLAIGLIVGANILAAGPFSGGS | 220 |
| SoPIP2-1 | 168 | EIIGTFVLVYTVFSATDPK-RSARDS------HVPILAPLPIGFAVFMVHLATIPITGTG | 220 |
| ZmPIP1-1 | 183 | EIVGTFILVYTVFSATDAK-RNARDS------HVPILAPLPIGFAVFLVHLATIPITGTG | 235 |
| ZmPIP1-2 | 184 | EIVGTFILVYTVFSATDAK-RNARDS------HVPILAPLPIGFAVFLVHLATIPITGTG | 236 |
| ZmPIP1-3 | 187 | EIVGTFILVYTVFSATDAK-RNARDS------HVPILAPLPIGFAVFLVHLATIPITGTG | 239 |
| ZmPIP1-5 | 183 | EIVGTFVLVYTVFSATDAK-RSARDS------HVPILAPLPIGFAVFLVHLATIPITGTG | 235 |
| ZmPIP2-1 | 179 | EIIGTFVLVYTVFSATDPK-RNARDS------HVPVLAPLPIGFAVFMVHLATIPVTGTG | 231 |
| ZmPIP2-2 | 181 | EIVGTFVLVYTVFSATDPK-RNARDS------HVPVLAPLPIGFAVFMVHLATIPVTGTG | 233 |
| ZmPIP2-3 | 179 | EIIGTFVLVYTVFSATDPK-RSARDS------HVPVLAPLPIGFAVFMVHLATIPITGTG | 231 |
| ZmPIP2-4 | 178 | EIIGTFVLVYTVFSATDPK-RSARDS------HVPVLAPLPIGFAVFMVHLATIPITGTG | 230 |
| ZmPIP2-5 | 174 | EIIGTFVLVYTVFSATDPK-RNARDS------HVPVLAPLPIGFAVFMVHLATIPITGTG | 226 |
| ZmPIP2-6 | 178 | EIIGTFVLVYTVFSATDPK-RNARDS------HVPVLAPLPIGFAVFMVHLATIPITGTG | 230 |
| ZmPIP2-9 | 176 | EIVGTFVLVYTVFSATDPK-RNARDS------HVPVLAPLPIGFAVFMVHLATIPITGTG | 228 |
| ZmPIP2-8 | 170 | EIVGTFVLVYTVFSATDPK-RKVRDS------HVPVLAPLPIGFAVFMVHLATIPVTGTG | 222 |
| ZmPIP2-7 | 174 | EIVGTFILVYTVFSATDPK-RTARDS------FIPVLVPLPIGFAVFVVHLATIPITGTG | 226 |
| ZmTIP1-1 | 147 | EIVMTFGLVYTVYATAVDP-K---KG------SLGTIAPIAIGFIVGANILVGGAFDGAS | 196 |
| ZmTIP1-2 | 148 | EMVMTFGLVYTVYATAVDP-K---KG------DLGVIAPIAIGFIVGANILAGGAFDGAS | 197 |
| ZmTIP2-1 | 145 | EVVITFALVYTVYATAADP-K---KG------SLGTIAPIAIGFIVGANILAAGPFSGGS | 194 |
| ZmTIP2-2 | 146 | EIVITFALVYTVYATAADP-K---KG------SLGTIAPIAIGFIVGANILAAGPFSGGS | 195 |
| ZmTIP2-3 | 145 | EIVITFALVYTVYATAADP-K---KG------SLGTIAPMAIGFIVGANILAAGPFSGGS | 194 |
| ZmTIP2-4 | 145 | EIVITFALVYTVYATAADP-K---KG------SLGTIAPIAIGFIVGANILAAGPFSGGS | 194 |
| ZmTIP3-1 | 152 | EAVMTFGLMYAYYATVIDP-K---RG------HVGTIAPLAVGFLLGANVLAGGPFDGAG | 201 |
| ZmTIP3-3 | 152 | EAAMAFGLMYAYFATVMDKARRVRAG------AGALAAPLAVGLLAGANVLACGALEGAV | 205 |
| ZmTIP3-2 | 157 | EAVMTFGLVYAYYATVVDP-KRG---------HLGTIAPLAVGFLLGANVLAGGPFDGAG | 206 |
| ZmTIP3-4 | 151 | EAVMTFGLMYAVYATAVDH-R-SRGG------AVA-IAPLAIGFVLGANILAGGPFDGAA | 201 |
| ZmTIP4-1 | 152 | EVILTFSLLFVTYAMILDP-R----S------QVRAIGPLLTGLIVGANSLAGGNFTGAS | 200 |
| ZmTIP4-2 | 208 | EVILTFSLLFVTYAMILDP-RS----------QVRTIGPLLTGLIVGANSLAGGNFTGAS | 256 |
| ZmTIP4-3 | 145 | EAVFTFSLLFVIYATILDP-R----K------LLPGAGPLLTGLLVGANSVAGAALSGAS | 193 |
| ZmTIP4-4 | 147 | EAVLTFSLLFAVYATVVDP-R----R------AVGGMGPLLVGLVVGANVLAGGPFSGAS | 195 |
| ZmTIP5-1 | 148 | EGVLTFLLVYTVHVVGERE-PRSRGGDGKREFAATALGALAVGLTQGAFVLAAGALTGAS | 206 |
| ZmNIP1-1 | 166 | EIITTFYLMFVISGVATDN-R-----------AIGELAGLAVGATILLNVLIAGPVSGAS | 213 |
| ZmNIP1-3 | 172 | EFIISFNLMFVVSGVATDN-R-----------AIGELAGLAVGATVLLNVLFAGPISGAS | 219 |
| ZmNIP1-4 | 177 | EFIITFYLMFVVMAVATDD-R-----------AVGQMAGLAVGGTIMLNALFAGPVSGAS | 224 |
| ZmNIP2-1 | 168 | EIIVTFNMMFVTLAVATDT-R-----------AVGELAGLAVGSAVCITSIFAGAVSGGS | 215 |
| ZmNIP2-4 | 170 | EVIVTFNMMFVTLAVATDT-R-----------AVGELAGLAVGSAVCITSIFAGAVSGGS | 217 |
| ZmNIP2-2 | 173 | EIVVTFNMMFVTCAVATDS-R-----------AVGELAGLAVGSAVCITSIFAGPVSGGS | 220 |
| ZmNIP2-3 | 176 | EIVVTFNMMFVTCAVATDS-R-----------AVGELAGLAVGSAVCITSIFAGPVSGGS | 223 |
| ZmNIP3-1 | 197 | EFIISFNLLFVVTAVATDT-R-----------AVGELAGIAVGAAVTLNILVAGPTTGGS | 244 |
| ZmNIP3-2 | 185 | ELVLTFVLMFVIAAVATDP-T-----------SSKELVAIAIAAAIMMNALIGGPSTGPS | 232 |
| ZmNIP4-1 | 175 | ELVASALLMVVIATAARG--------------SNQTAGGLAIGAAVGALGLVIGPVSGGS | 220 |
| ZmSIP1-1 | 142 | EGVLTFVITLTVLWVIVKG-P-----------RNVILKTLLLSTSIVSVILAGAEYTGPS | 189 |
| ZmSIP1-2 | 140 | ELVLTFVITLAVLLIIVKG-P-----------RNPIIKTWMISICTLCLVLSGAAYTGPS | 187 |
| ZmSIP2-1 | 140 | EGLATFMVVMVSVTLKKKE-M-----------KSFFMKTWITSIWKNTIHLLSSDITGGI | 187 |

```
                     HE                TM6
AtTIP2-1   221  MNPARSFGPAVA--A-GDFSGHWVYWVGPLIGGGLAGLIYGNVFMGSSEHV------PLA  271
SoPIP2-1   221  INPARSFGAAVIFNSNKVWDDQWIFWVGPFIGAAVAAAYHQYVLRAAAIKALG----SFR  276
ZmPIP1-1   236  INPARSLGAAVIYNQHHAWADHWIFWVGPFIGAALAAIYHQVIIRAI----------PFK  285
ZmPIP1-2   237  INPARSLGAAIIYNRDHAWNDHWIFWVGPFIGAALAAIYHQVIIRAI----------PFK  286
ZmPIP1-3   240  INPARSLGAAIIYNRDHAWSDHWIFWVGPFIGAALAAIYHQVIIRAI----------PFK  289
ZmPIP1-5   236  INPARSLGAAIVYNRSHAWNDHWIFWVGPFIGAALAAIYHVVIIRAL----------PFK  285
ZmPIP2-1   232  INPARSLGAAVIYNKDKPWDDHWIFWVGPLVGAAIAAFYHQYILRAGAIKALG----SFR  287
ZmPIP2-2   234  INPARSLGAAVVYNKDKPWDDHWIFWVGPLLGAAIAAFYHQYILRAGAIKALG----SFR  289
ZmPIP2-3   232  INPARSLGAAVIYNKDKAWDDQWIFWVGPLIGAAIAAAYHQYVLRASATKLG-----SYR  286
ZmPIP2-4   231  INPARSLGAAVIYNKDKAWDDQWIFWVGPLIGAAIAAAYHQYVLRASATKLG-----SYR  285
ZmPIP2-5   227  INPARSLGAAVIYNNDKAWDDHWIFWVGPFIGAAIAAAYHQYVLRASAAKLG-----SSA  281
ZmPIP2-6   231  INPARSLGAAVVYNNSKAWSDQWIFWVGPFIGAAIAALYHQIVLRASARGYG-----SFR  285
ZmPIP2-9   229  INPARSLGAAVVYNNSKAWSDQWIFWVGPFIGAAIAALYHQIVLRASARGYG-----SFR  283
ZmPIP2-8   223  INPARSLGPAVVYNQRKAWEDHWIFWVGPLIGAAAAMLYHQLVLRAGAAKAFA----SFR  278
ZmPIP2-7   227  INPARSLGAAVLYNQHDAWKDHWIFWVGPLIGATVAALYHKLVLRGEAVKALG----SFR  282
ZmTIP1-1   197  MNPAVSFGPALV--S-WEWGYQWVYWVGPLIGGGLAGVIYELLFISHTHE-------QLP  246
ZmTIP1-2   198  MNPAVSFGPAVV--T-GVWENHWVYWVGPLAGAAIAALVYDIIFIGQRPHQQL----PTT  250
ZmTIP2-1   195  MNPARSFGPAVA--A-GDFAGNWVYWVGPLVGGGLAGLVYGDVFIGGSYQ-------QVA  244
ZmTIP2-2   196  MNPARSFGPAVA--A-ADFAGNWVYWVGPLIGGGLAGLVYGDVFIGGSYQ-------QVA  245
ZmTIP2-3   195  MNPARSFGPAVA--A-GNFAGNWVYWVGPLVGGGLAGLVYGDVFIASYQ--------PVG  243
ZmTIP2-4   195  MNPARSFGPAVA--A-GNFAGNWVYWVGPLIGGGLAGLVYGDVFIASYQ--------PVG  243
ZmTIP3-1   202  MNPARVFGPALV--G-WRWRHHWVYWLGPFLGAGLAGLVYEYLVIPSADAAVPHAHQPLA  258
ZmTIP3-3   206  MNPARAFGPAVV--GSRRWRHQWVYWVGPMVGAGLSGVVYEHLVAGPAAEEEE----PAP  259
ZmTIP3-2   207  MNPARVFGPALV--G-WRWRHHWVYWLGPFLGAGLAGLVYEYLLIPPADAVPHTHQ-PLA  262
ZmTIP3-4   202  MNPARAFGPALV--G-WSWRHHWVYWVGPLIGAGLAGGLYEFVMVEQEPEAPAPAAVPRM  258
ZmTIP4-1   201  MNPARSFGPALA--T-GDWTNHWVYWIGPLLGGPLAGFVYESLFLVQKMHE------PLL  251
ZmTIP4-2   257  MNPARSFGPAMA--T-GVWTNHWVYWIGPLLGGSLAGFVYESLFMVYKTHE------PLL  307
ZmTIP4-3   194  MNPARSFGPAVA--S-GIWTHHWVYWVGPLAGGPLAVLVYECCFIAAAPTHA-----LLP  245
ZmTIP4-4   196  MNPARSFGPALV--A-GVWADHWVYWVGPLIGGPLAGLVYDGLFMAQGGHEPL----PRD  248
ZmTIP5-1   207  MNPARSFGPAVV--S-GHFKNQAVYWAGPMVGAAVAALVYQIMACPSVTG-------NVE  256
ZmNIP1-1   214  MNPARSVGPALV--S-GEYTSIWVYVVGPVVGAVAGAWAYNLIRFTNKPLREITKSTSFL  270
ZmNIP1-3   220  MNPARTLGPALV--V-GRYAGIWVYFAGPICGTVAGAWAYNLIRFTDKPLREITQTSSFL  276
ZmNIP1-4   225  MNPARSIGPALV--S-NKFRALWVYIFGPFAGAAAGAWAYNLIRHTDKTLAEVTKSASQT  281
ZmNIP2-1   216  MNPARTLGPALA--S-NLYTGLWIYFLGPVLGTLSGAWTYTYIRFEEAPSHKDM---SQK  269
ZmNIP2-4   218  MNPARTLGPALA--S-NLYTGLWIYFLGPVLGTLSGAWTYTFIRFEEAPSKDASSSHSQK  274
ZmNIP2-2   221  MNPARTLAPAVA--S-NVFTGLWIYFLGPVIGTLSGAWVYTYIRFEEAPAAKD----TQR  273
ZmNIP2-3   224  MNPARTLAPAVA--S-NVFTGLWIYFLGPVVGTLSGAWVYTYIRFEEAPAAAK--PDTQR  278
ZmNIP3-1   245  MNPVRTLGPAVA--A-GNYRQLWIYLLAPTLGALAGASVYKAVKLRDENGETPRTQRSFR  301
ZmNIP3-2   233  MNPARTIGAALA--T-GKYKDIWVYLLAPPLGAIAGAATYTLIKP--------------- 274
ZmNIP4-1   221  MNPIRTLGPAIV--L-GRYTSVWIYLVAPVAGMLIGALCNRLVRRSDAIIAFLCGAKPRV  277
ZmSIP1-1   190  MNPANAFGWAYVNNWHNTWEQLYVYWICPFIGAMLAGWIFRVVFLPPAPK-------PKT  242
ZmSIP1-2   188  MNPANAFGWAYVNNRHNTWEQFYVYWICPFIGAILAAWIFRAMFLTPPPK-------PKA  240
ZmSIP2-1   188  MNPASAFAWAYARGDHTTFDHLLVYWLAPLQATLLGVWAVTFFTKPKKIKEQKVDENKIK  247
```

```
AtTIP2-1   272   SADF-------------------------   275
SoPIP2-1   277   SNPTN------------------------   281
ZmPIP1-1   286   SRS--------------------------   288
ZmPIP1-2   287   SRS--------------------------   289
ZmPIP1-3   290   SRS--------------------------   292
ZmPIP1-5   286   SRD--------------------------   288
                 *
ZmPIP2-1   288   SNA--------------------------   290
                 *
ZmPIP2-2   290   SNA--------------------------   292
ZmPIP2-3   287   SNA--------------------------   289
ZmPIP2-4   286   SNA--------------------------   288
ZmPIP2-5   282   SFSR-------------------------   285
ZmPIP2-6   286   SNA--------------------------   288
ZmPIP2-9   284   SNA--------------------------   286
ZmPIP2-8   279   NNQHF------------------------   283
ZmPIP2-7   283   STSATV-----------------------   288
ZmTIP1-1   247   STDY-------------------------   250
ZmTIP1-2   251   AADY-------------------------   254
ZmTIP2-1   245   DQDYA------------------------   249
ZmTIP2-2   246   DQDYA------------------------   250
ZmTIP2-3   244   QQEYP------------------------   248
ZmTIP2-4   244   QQEYP------------------------   248
ZmTIP3-1   259   PEDY-------------------------   262
ZmTIP3-3   260   SCGDRRRA---------------------   267
ZmTIP3-2   263   PEDY-------------------------   266
ZmTIP3-4   259   PVASEDY----------------------   265
ZmTIP4-1   252   NGEV-------------------------   255
ZmTIP4-2   308   NGDI-------------------------   311
ZmTIP4-3   246   QQDP-------------------------   249
ZmTIP4-4   249   DTDF-------------------------   252
ZmTIP5-1   257   AVVV-------------------------   260
ZmNIP1-1   271   KSTSRMNSAASA-----------------   282
ZmNIP1-3   277   RSVRRSSS---------------------   284
ZmNIP1-4   282   NAS--------------------------   284
ZmNIP2-1   270   LSSFKLRRLQSQSVAVDDDELDHIQV---   295
ZmNIP2-4   275   LSSFKLRRLQSQSVAADADDDEELDHIQV   303
                           #
ZmNIP2-2   274   LSSFKLRRMQSQLAADEFDTV--------   294
ZmNIP2-3   279   LSSFKLRRMQSQSALAADEFDTV------   301
ZmNIP3-1   302   R----------------------------   302
ZmNIP3-2   275   -----------------------------   274
ZmNIP4-1   278   VAPGQNRAARRWSTCVSALLAG-------   299
ZmSIP1-1   243   KKA--------------------------   245
ZmSIP1-2   241   KKA--------------------------   243
ZmSIP2-1   248   KE---------------------------   249
```

Supplemental Figure S1. Alignment of important domains of ZmAQPs and the predicted phosphorylation sites. Multiple sequence alignment was performed using the MAGE-X MUSCLE algorithm. Dashes indicate gaps in amino acid sequences. Numbers represent the distance from the protein's translation start site. The conserved domains and amino acid residues are highlighted in different colours. The gray boxes indicate the transmembrane helices (TM1–TM6) and the two short helices (HB and HE). P1–P5 residues (shown in green), NPA motifs (shown in blue), and the ar/R selectivity filter (shown in red) are shown. The identified aquaporins AtTIP2-1 (At3g16240) and SoPIP2-1 (SOVF_195660) were used as the control. Phosphorylation sites predicted by the online website (NetPhos 2.0) are highlighted in yellow. Experimentally confirmed phosphorylation sites are shown in orange font and marked with * and #.

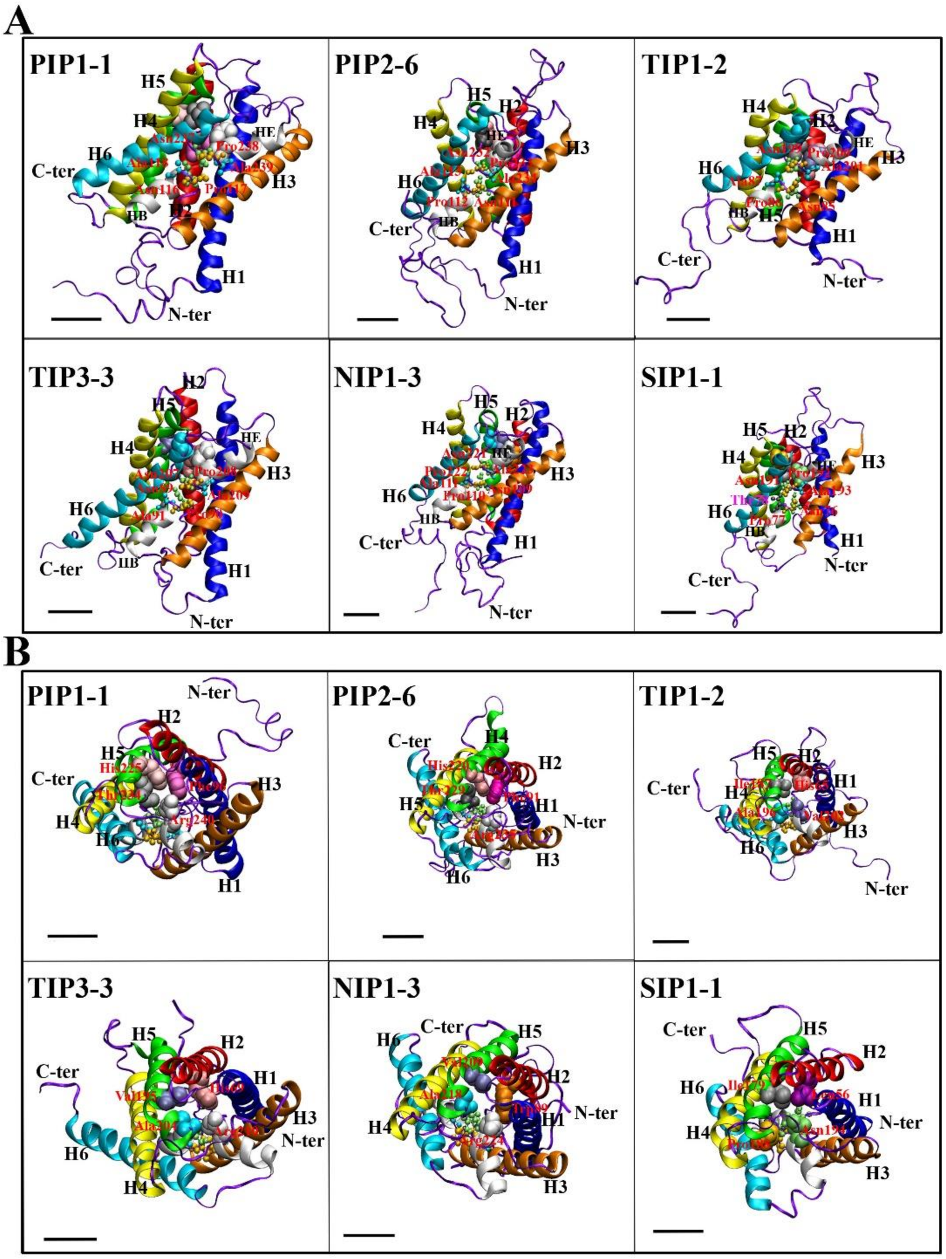


**Supplemental Figure S2**. 3D protein structure of ZmAQPs. 3D structure was predicted by phyre 2.0 and preformed with VMD. Top view (A) and side view (B) of 3D protein structure. Each protein molecular model consists of six transmembrane α-helices (H1-H6) and two short α-helices (HB and HE). Transmembrane α-helices are indicated in different colors with NewCarton (H1, blue; H2, red; H3, Orange; H4, Yellow; H5, Green; H6, Cyan2; HB and HE,White). Purple NewRibbons present the remaining part. Two NPA motifs are located in HB and HE and displayed in CPK pattern. The selectivity filter residues (H2, H5, LE1, LE2) are shown as VDW. Amino acids involved in the picture are in different colors (Asn, Lime; Pro, Orange3; Ala, Cyan2; Thr, gray; Phe, Magenta2; His, Pink; Arg, White; Ile, Silver; Val, iceblue; Trp, Orange; Leu, purple;). N-ter and C-ter indicate amino-terminal and carboxi-terminal regions, respectively. Scale bars in the figures indicate 10 Å.

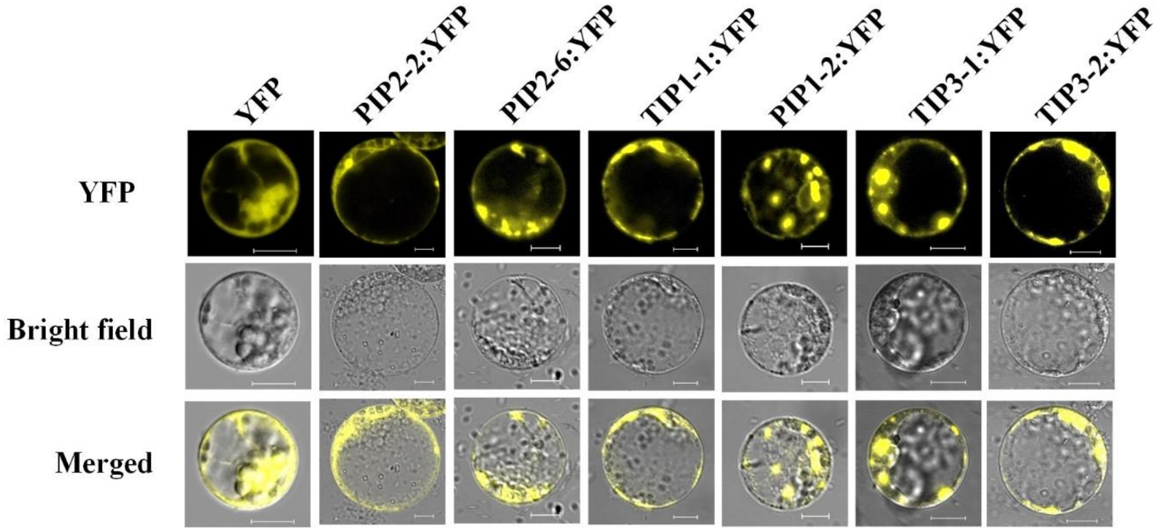


**Supplemental Figure S3**. Protoplast transient expression analysis using ZmAQP-YFP fusion constructs. Subcellular localization of ZmAQPs were determined in maize protoplasts. Mesophyll protoplasts of seedling transient expression of ZmAQP-YFP. Yellow signals indicate fluorescence emitted by YFP. Scale bars indicate 10 μM.

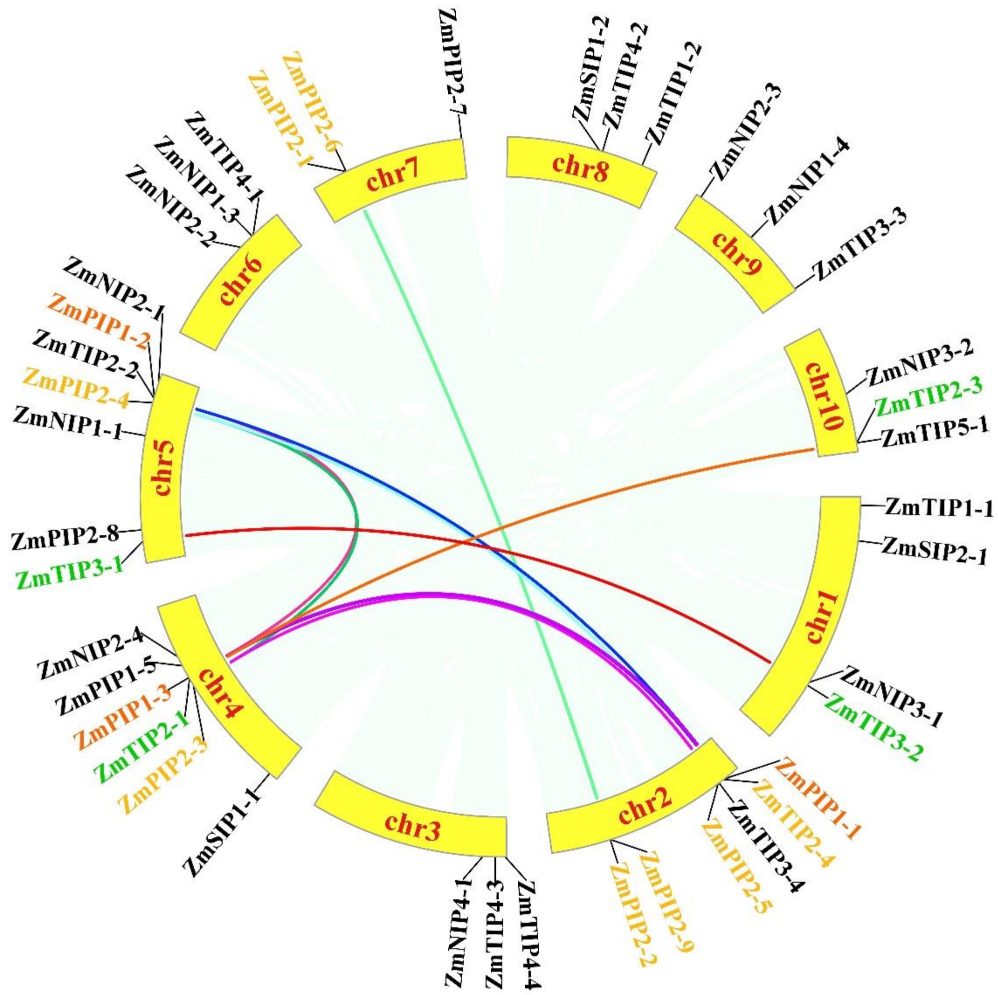


**Supplemental Figure S4**. Chromosomal distribution and synteny analysis of *ZmAQP* genes. The chromosomal location and gene duplication were analyzed by Mapinspect and TBtools (MCScan) software. The colored lines indicate the segmental duplication AQP gene pairs. The chromosome number is illustrated on the chromosomes (chr).

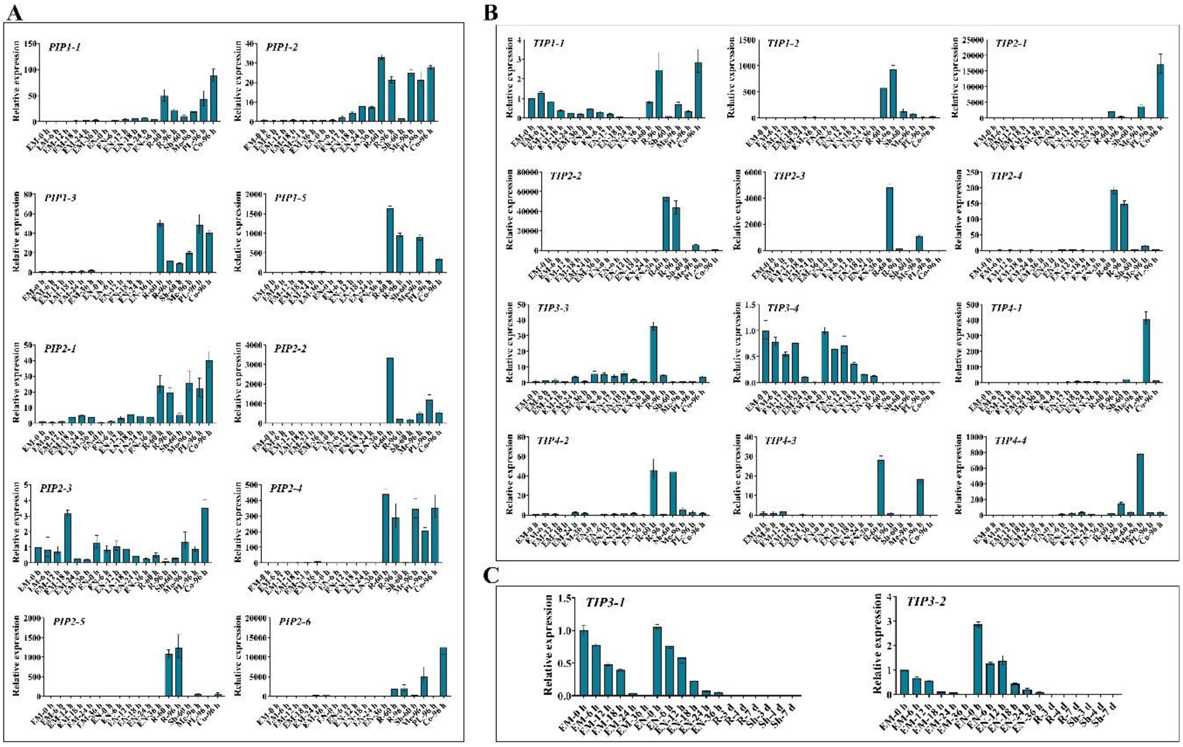


**Supplemental Figure S5**. qRT-PCR analysis of the expression levels of *ZmAOPs* during early seed germination. The relative transcript levels of *ZmPIPs* (A) and Zm*TIPs* (B-C) in Z58 during the seed germination process. In total 18 samples from seeds and seedlings at different times after seed imbibition were used in the assay. *ACTIN 1* was used as an internal control. Data are means ± *SD* (n = 3). Three independent experiments produced similar results. EM, embryo; EN, endosperm; R, root; Sh, shoot; Me, mesocotyl; PL, plumule; Co, coleoptile.

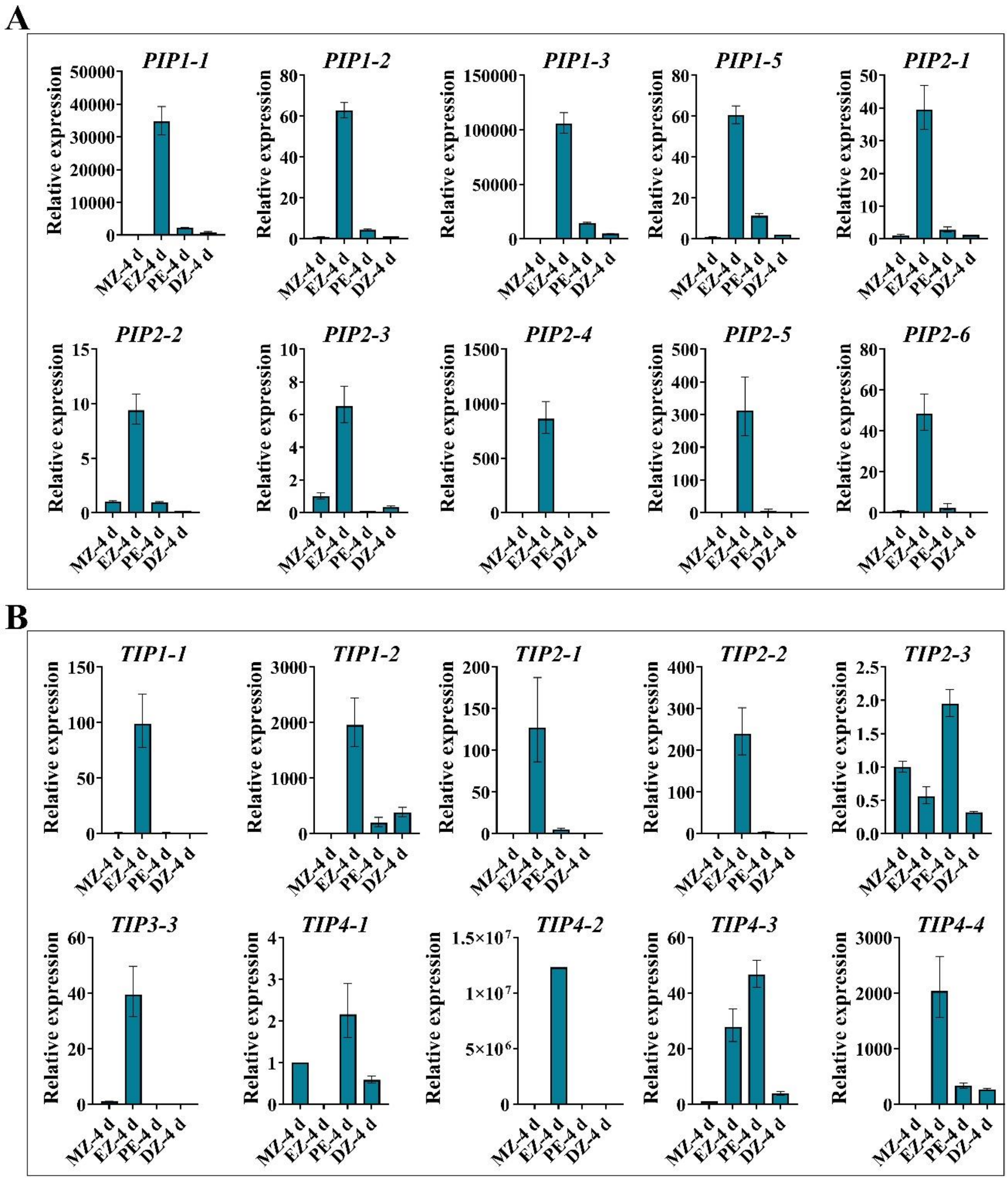


**Supplemental Figure S6.** RT-qPCR analysis of the expression levels of *ZmAOPs* in different zones of the seedling root. The relative transcript levels of *ZmPIPs* (A) and Zm*TIPs* (B) in Z58. Samples from different zones of the primary root at 4 days after seed imbibition were used in the assay. *ACTIN 1* was used as an internal control. Data are means ± *SD* (n = 3). MZ-root cap and meristem zone; EZ-elongation zone; PZ-post elongation zones; DZ-differentiation zone.

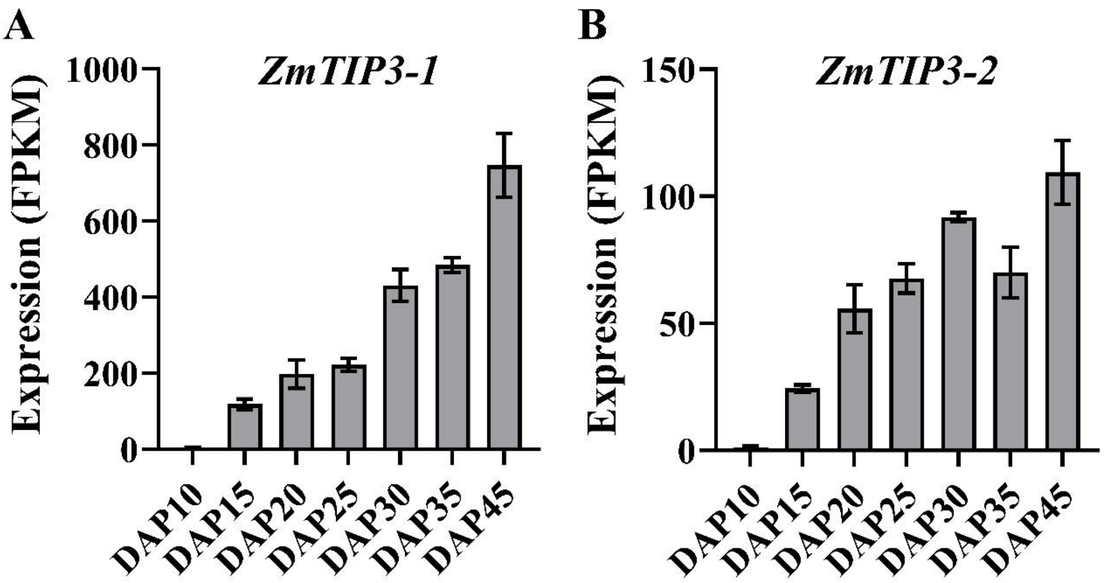


**Supplemental Figure S7**. Expression profile of *ZmTIP3s* during seed development. Gene expression analysis was obtained by RNA-sequencing. FPKM values of *ZmTIP3-1* (A) and *ZmTIP3-2* (B) were evaluated 10–45 days after pollination (DAP10 to DAP45). Data are the means of three biological replicates.

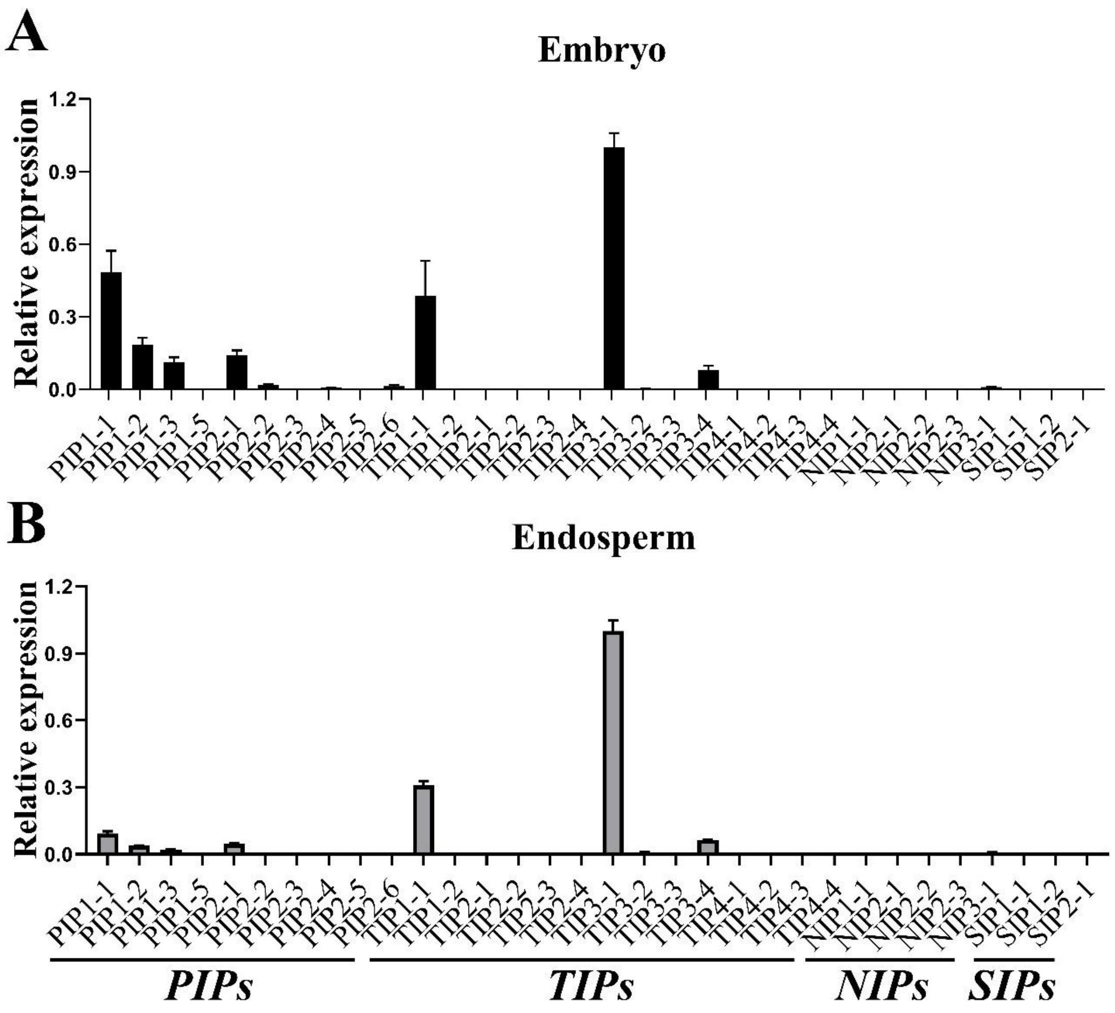


**Supplemental Figure S8.** qRT-PCR analysis of the expression of *ZmAOPs* in dry seed. The relative transcript levels of *ZmAQPs* in the embryo (A) and endosperm (B) in dry B73 seeds. *ACTIN 1* was used as an internal control. Data are means ± *SD*.

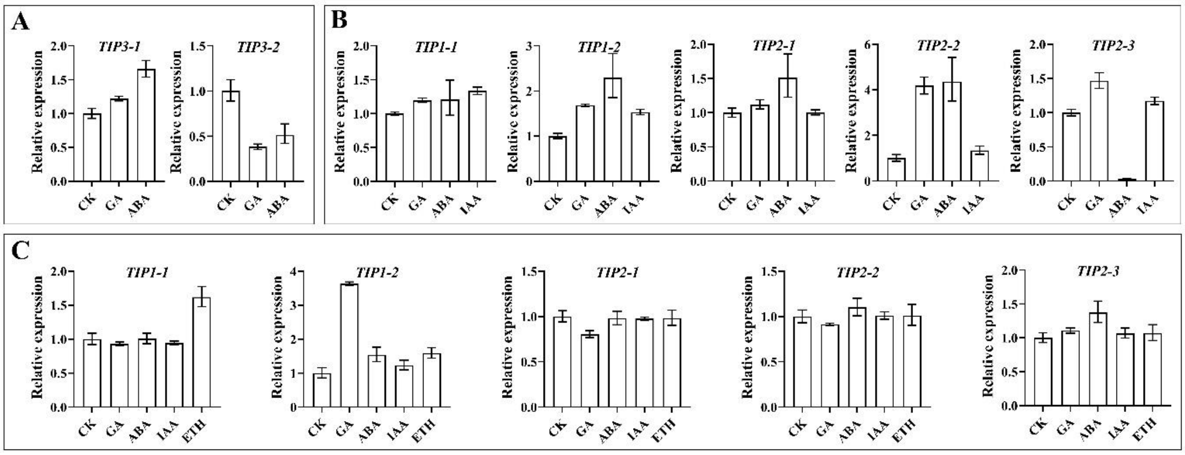


**Supplemental Figure S9**. The expression of *ZmAQPs* in response to plant hormones. Three groups of treatments were carried out. (A) dry seeds were imbibed in the solutions of 10 ABA and GA; ABA, GA, IAA, and Eth were spared on the germinated seeds (immediately after radicle protrusion) (B) and seedlings (C), respectively. Water treatment was used as a control. All the samples for analysis were collected three hours after treatment. *ACTIN 1* was used as an internal control. Data are means ± *SD*.

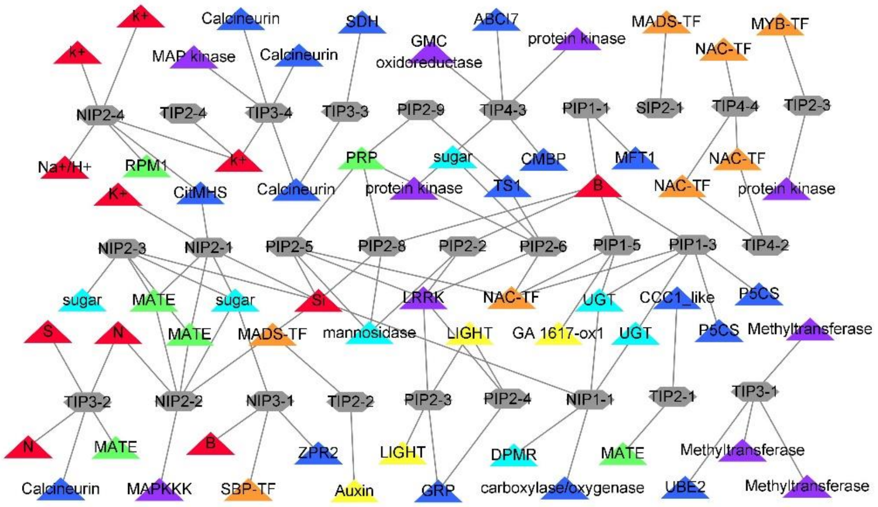


**Supplemental Figure S10**. Protein-Protein interaction (PPI) network of ZmAQPs. The retrieval of interacting proteins database (STRING v11.0) was used to construct the PPI network. Grey hexagon: ZmAQPs; Red triangle: the protein relative to transport; orange triangle: all kinds of transcription factor; yellow triangle: protein relative with the Auxin and light; green triangle: protein relative to resist pathogen; cyan triangle: relative to the sugar; purple triangle: relative to the protein kinase and the methyl-transferase. blue triangle: no specific classify proteins.

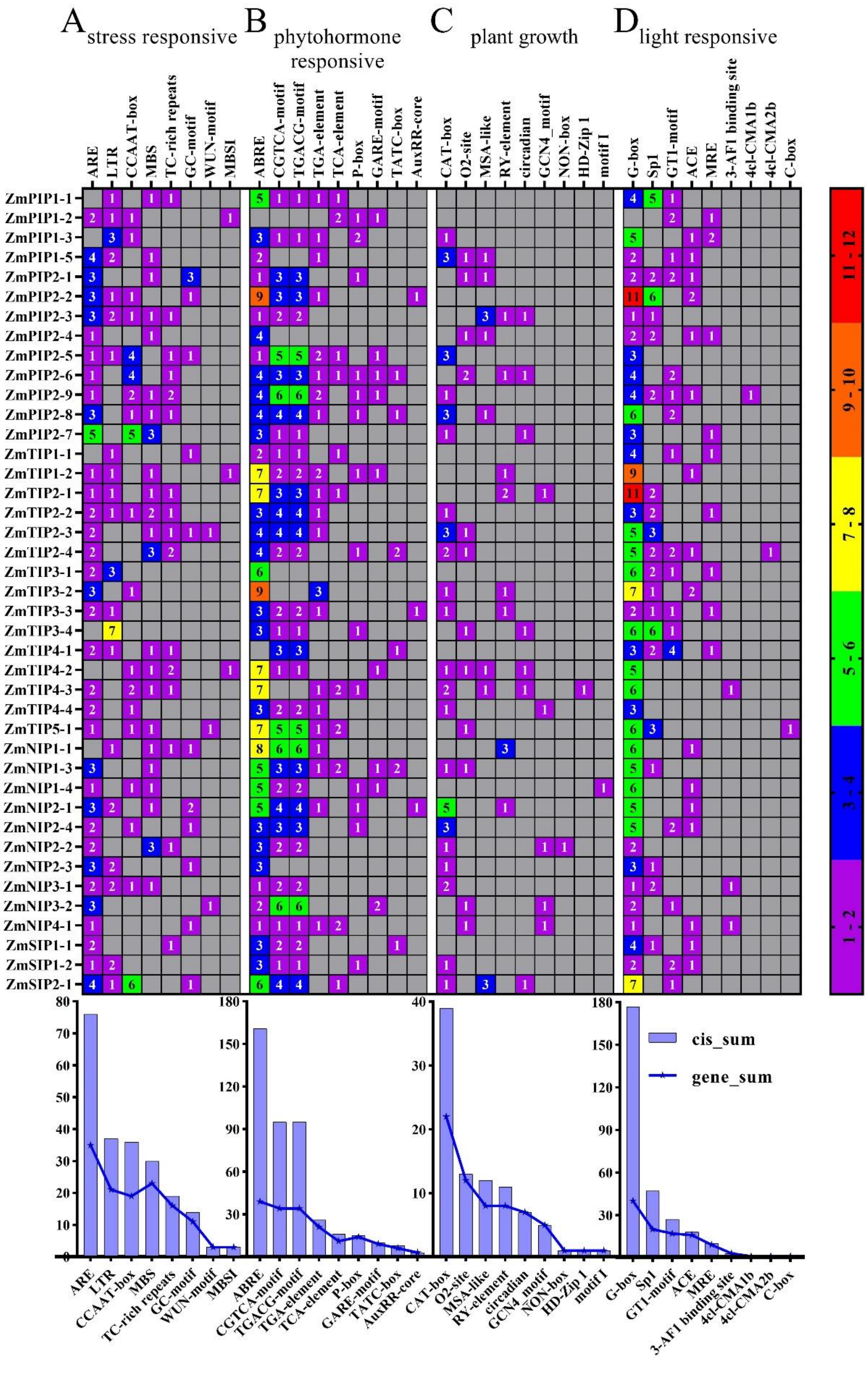


**Supplemental Figure S11.** Various cis-acting elements in the promoters of *ZmAQP* genes. (A) The distribution of cis-acting elements involved in stress response (A), phytohormone response (B), plant growth-related (C), and light response (D) and their amounts in ZmAQP promoters.
